# Relative Risk of Visceral Leishmaniasis in Brazil: A Spatial Analysis in Urban Area

**DOI:** 10.1371/journal.pntd.0002540

**Published:** 2013-11-07

**Authors:** Valdelaine Etelvina Miranda de Araújo, Letícia Cavalari Pinheiro, Maria Cristina de Mattos Almeida, Fernanda Carvalho de Menezes, Maria Helena Franco Morais, Ilka Afonso Reis, Renato Martins Assunção, Mariângela Carneiro

**Affiliations:** 1 Laboratório de Epidemiologia de Doenças Infecciosas e Parasitárias, Departamento de Parasitologia, Instituto de Ciências Biológicas, Universidade Federal de Minas Gerais, Belo Horizonte, Brazil; 2 Secretaria Municipal de Saúde, Prefeitura de Belo Horizonte, Belo Horizonte, Minas Gerais, Brazil; 3 Departamento de Estatística, Instituto de Ciências Exatas, Universidade Federal de Minas Gerais, Belo Horizonte, Brazil; 4 Centro de Pesquisa René Rachou, Fundação Oswaldo Cruz, Belo Horizonte, Brazil; 5 Departamento de Ciência da Computação, Instituto de Ciências Exatas, Universidade Federal de Minas Gerais, Belo Horizonte, Brazil; 6 Pós-graduação em Infectologia e Medicina Tropical, Faculdade de Medicina, Universidade Federal de Minas Gerais, Belo Horizonte, Brazil; National Institutes of Health, United States of America

## Abstract

**Background:**

Visceral leishmaniasis (VL) is a vector-borne disease whose factors involved in transmission are poorly understood, especially in more urban and densely populated counties. In Brazil, the VL urbanization is a challenge for the control program. The goals were to identify the greater risk areas for human VL and the risk factors involved in transmission.

**Methodology:**

This is an ecological study on the relative risk of human VL. Spatial units of analysis were the coverage areas of the Basic Health Units (146 small-areas) of Belo Horizonte, Minas Gerais State, Brazil. Human VL cases, from 2007 to 2009 (n = 412), were obtained in the Brazilian Reportable Disease Information System. Bayesian approach was used to model the relative risk of VL including potential risk factors involved in transmission (canine infection, socioeconomic and environmental features) and to identify the small-areas of greater risk to human VL.

**Principal Findings:**

The relative risk of VL was shown to be correlated with income, education, and the number of infected dogs per inhabitants. The estimates of relative risk of VL were higher than 1.0 in 54% of the areas (79/146). The spatial modeling highlighted 14 areas with the highest relative risk of VL and 12 of them are concentrated in the northern region of the city.

**Conclusions:**

The spatial analysis used in this study is useful for the identification of small-areas according to risk of human VL and presents operational applicability in control and surveillance program in an urban environment with an unequal spatial distribution of the disease. Thus the frequent monitoring of relative risk of human VL in small-areas is important to direct and prioritize the actions of the control program in urban environment, especially in big cities.

## Author Summary

Visceral leishmaniasis (VL) is a vector-borne disease whose factors involved in transmission are poorly understood, especially in more urban and densely populated counties. In Brazil, the increasing occurrence of human VL cases in urban centers is a challenge for the control program. We aimed to identify the risk areas for VL and the risk factors involved in transmission in Belo Horizonte, a large urban area of the Brazil. At the same geographical space, we analyzed human VL cases (n = 412), canine infection and socioeconomic and environmental features. We identified a concentration of high-risk small-areas of human VL cases in the northern part of the city, marked by worse levels of education and income, and higher number of infected dogs per inhabitants. The spatial analysis used is useful for the identification of small-areas with a greater risk of VL and displays operational applicability in the control program in an urban environment with an unequal spatial distribution of the disease. Thus, the frequent monitoring of risk of human VL according to small-areas is important to direct and prioritize the actions of the control program in urban environment, especially in big cities.

## Introduction

Visceral leishmaniasis (VL) is a vector-borne disease highly influenced by social and environmental factors. The majority (>90%) of cases is concentrated in six countries: Bangladesh, Brazil, Ethiopia, India, Nepal and Sudan [1]. In Brazil, the VL is caused by *Leishmania infantum*, belonging to the *Leishmania donovani* complex that is mainly transmitted by the sand fly *Lutzomyia longipalpis*. Dogs are considered to be the principal parasite reservoir, playing an important role in the transmission cycle in urban areas. The VL urbanization has been documented since the 1980s [2]–[8]. This trend represents a challenge for the control of the disease in urban areas [6]–[7], [9].

The average incidence rate of VL in Brazil was 1.9/100,000 inhabitants between 1994 and 2009 [10]. During the last decades, an increasing number of clinical VL cases have been reported for large Brazilian cities, including Belo Horizonte. In 1994, the first human VL cases occurred in BH (n = 29), with incidence rate of 1.4 cases/100,000 inhabitants and case fatality rate of 20.7%. From 1994 to 2009, the highest incidence rate was 7.2/100,000 (in 2008), the case fatality rate 23.6% (in 2009) and the proportion of infected dogs was 9.9% (in 2006) [11].

The presence of the vector and of the canine reservoir has been described throughout Belo Horizonte with an unequal geographic distribution [11]–[15], which is likely due to the intra-urban differences present in large cities. In Belo Horizonte, the Health Vulnerability Index incorporates intra-urban differences represented by indicators of sanitation, housing, income, education, and health [16]. The Health Vulnerability Index is useful in the identification of areas with unfavorable socioeconomic conditions, which are priorities for interventions and the allocation of resources for public policies.

In Brazil, the Visceral Leishmaniasis Control and Surveillance Program (VLSCP) is based on reducing the morbidity and case-fatality rates through the early diagnosis and treatment of human cases and on decreasing the transmission risk by controlling the population of both domestic reservoirs and the vector [9]. However, in urban areas, the VLSCP has encountered difficulties, including: logistics and a high cost for a chemical control of the vector; insufficient environmental management for vector control; large interval between diagnosis and the elimination of infected dogs; dissatisfaction among the human population with the elimination of infected dogs; insufficient accuracy of the tests to detect infection, thereby allowing asymptomatic dogs to persist as a source of infection for the vector; substitution of culled dogs by a new susceptible canine population; and high canine infection and infectiveness rates [8], [17]–[19].

The spread of the disease in Belo Horizonte has occurred despite systematic interventions since 1994, which reflects the difficulty of control in urban areas. Thus, the identification of areas with a greater disease risk may help to direct and prioritize the actions of the VLSCP. This study aimed to identify the greater risk areas for human VL and the risk factors involved in transmission, using spatial analysis, in Belo Horizonte, Minas Gerais State, Brazil, during 2007–2009.

## Methods

### Ethics statement

This study was approved by the research ethics committees of the Universidade Federal de Minas Gerais-UFMG (No 211/09) and of the Belo Horizonte City Hall (No 075.2008). Given the assumptions of research ethics, we maintained the confidentiality of data during processing. Analyses were performed anonymously; hence the Informed Consent Form was not necessary.

### Study design

This is an ecological study on the relative risk of human VL, whose spatial units of analysis were the coverage areas of the Basic Health Units of Belo Horizonte.

### Study area

Belo Horizonte, capital of Minas Gerais State, has 2,375,151 inhabitants, and a population density of 7.2 inhabitants/km2. It is the sixth most populous city in Brazil according to the census of the Brazilian Institute of Geography and Statistics (Instituto Brasileiro de Geografia e Estatística-IBGE) [20]. The city is located at 852 meters above sea level, between latitude 19°49′01″S and longitude 43°57′21″W. It has a dry winter and a hot and rainy summer, with an average annual temperature of 21°C, average relative air humidity of 65%, and average annual rainfall of 1,500 mm [21].

**Figure 1:**
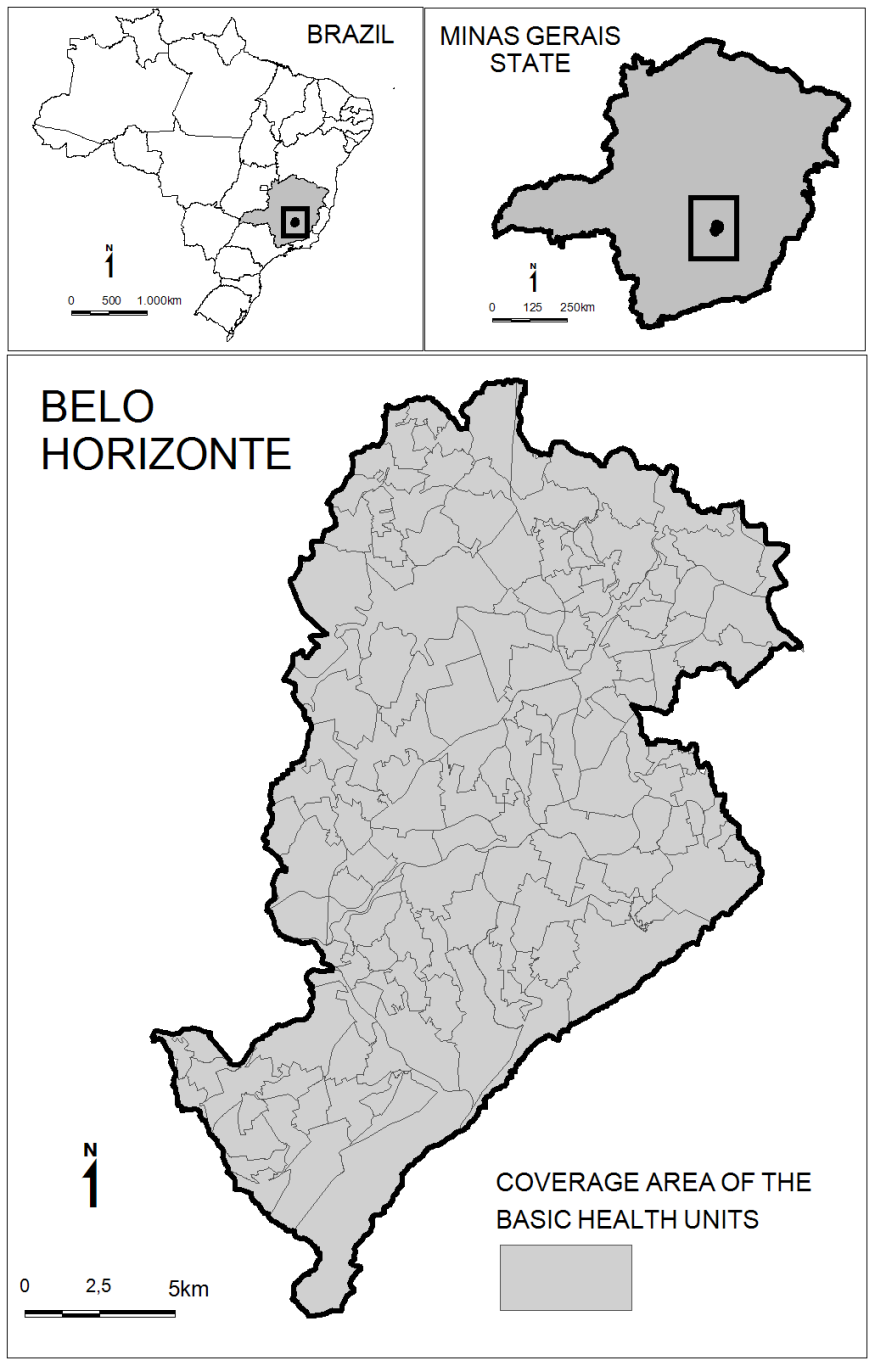
Map of Belo Horizonte (Brazil) with 146 coverage areas of the Basic Health Units.

In Belo Horizonte, the health services are organized territorially into nine health districts that are subdivided into 146 areas covered by Basic Health Units (Figure 1). These coverage areas are the result of aggregating 2,563 census tracts defined by the Brazilian Institute of Geography and Statistics (IBGE) [20]. The division between these coverage areas considers aggregation and geographical barriers, continuity occupation, transportation facilities and characteristics of homogeneity. It is noteworthy that the divisions are mainly administrative. The population of these areas varies from 2,197 to 45,171 inhabitants with an average of 15,331 inhabitants.

### Data

Human VL cases were selected with an onset of symptoms between 2007 and 2009 (n = 412), which were available in the Brazilian Reportable Disease Information System. The cases were georeferenced at the household level, which allowed identifying the respective coverage areas in which they were contained. During 2007–2009, the incidence rates were 4.9, 7.2 and 6.6/100,000 inhabitants and the case fatality rates were 8.2, 12.4 and 23.6%, respectively.

In Belo Horizonte, the domestic dog population was 301,593 dogs in 2009 (1 dog per 8 inhabitants). The actions of the Visceral Leishmaniasis Control and Surveillance Program related to the dogs are recorded in the Information System on Zoonosis Control. The canine blood samples are screened for antibodies against *Leishmania* by an enzyme-linked immunosorbent assay (ELISA) and confirmed by an indirect immunofluorescent antibody test (IFAT). In the studied period, 470,479 blood samples were tested, of which 7.8% were reactives. Some negative dogs were tested more than once. The data on canine infection were evaluated in two ways: number of infected dogs and number of infected dogs per inhabitants.

Among the components of the Health Vulnerability Index, we selected those that have been described in the literature as being associated with the occurrence of VL: indicators of urban services (water supply, sanitary sewage, and the destination of waste) and of the socioeconomic level (income and education). These data, available for the census tracts, were grouped according to the coverage areas by the average weighted by the population or by the number of households, depending on the main relation of the indicator.

We used a contour map of Belo Horizonte to calculate the average altitude of each area. The vegetation coverage was characterized by means of the Normalized Difference Vegetation Index (NDVI) [22]. The NDVI varied from −1 to +1. In general, the positive and negative values indicate the presence and absence of green vegetation, respectively. To calculate the NDVI, images were obtained by the Thematic Mapper sensor aboard the LANDSAT-5 satellite in July 2008, since this was the intermediate year in the analyzed period, and there were higher-quality images due to the relative absence of cloud cover that month. The images were adjusted to the spatial conformation of the coverage areas, and the minimum, maximum, mean, standard deviation, and median values of the NDVI were calculated.

The spatial statistical modeling used does not consider the time variable [23]. Therefore, we studied a short time series to minimize the potential effect of time in the estimation of the incidence rates. The period 2007–2009 was selected due to the availability of georeferenced data on dogs.

The analyses were performed using the MapInfo® 8.5 and WinBUGS 1.4 softwares and the geographical information system of Belo Horizonte.

### Descriptive analysis

The descriptive analyses were carried out in two steps. First, thematic maps were used to visualize the spatial distribution of cumulative incidence rates of human VL per 100,000 inhabitants (crude rates) and the ratio of infected dogs per 1,000 inhabitants in 146 coverage areas during 2007 to 2009. Second, scatter plots graphics were generated with the log-relative risk of VL (log-RR-VL) and the studied covariates. This process allowed for the selection of the covariates that were most correlated with the log-RR-VL for further statistical modeling.

### Spatial statistical modeling for the relative risks of VL

Data from neighboring areas were used to estimate the VL incidence rate of a certain area (smoothed rates) [23]. This approach was intended to minimize the instability in the incidence rates calculated for small-areas. Moreover, it produces more reliable estimates and smoother maps, which are easier to view and interpret from an epidemiological point of view [24]–[27]. As a definition for neighboring areas, we used the adjacency: areas which share borders were considered neighbors.

The dependent variable was the number of VL cases observed in area *i*, *O_i_*, *i* = 1,2,…,*N*, where *N* is the number of areas in the map. We assume that *O_i_* follows a Poisson distribution with mean λ_i_. Our aim was to model the relative risk 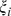, which is defined as 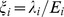, where *E_i_* is the expected number of VL cases in area *i* if all VL cases were homogeneously distributed over the entire map. To obtain a generalized linear model, the logarithmic link function was used: 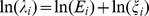. The covariates 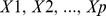 are incorporated into the model by 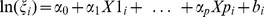, where 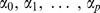 are coefficients to be estimated and *b_i_* is a random effect. Then,
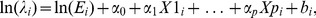
(1)where 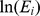 is an offset term.

To model the random effect *b_i_*, we used the convolution conditional autoregressive (CAR) model [23], which was defined by a set of conditional distributions. The random effect *b_i_* is decomposed into two components, 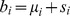, such that *μ_i_* are unstructured i.i.d. N(0,σ^2^_μ_) random effects. The component *s_i_* is spatially structured and follows an intrinsic conditional autoregressive model as follows:
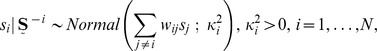
where *N* is the total number of areas in the map and 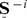 is the (*N-1*)-dimensional vector of observations in all *N* areas but the observation of area *i*. Let 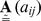 be a *N*×*N* neighborhood matrix so that 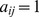 if the areas *i* and *j* are neighbors and 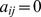 otherwise. By definition, 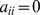. We define the matrix 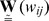 so that 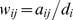, where 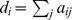 is the number of neighbors of area *i*. Each area must have at least one neighbor because islands are not allowed here. Therefore, 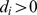. The parameter 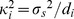 is the area *i* prior variance, and σ_s_^2^ is the common variance. Hence, a larger number of neighbors of an area imply a smaller prior variance.

It is worth noting that the expected value of *s_i_* takes into account not only information for area *i* but also information for its neighbors. In this sense, the approach [23] uses the spatial information in the neighborhood to estimate parameters related to each area of the map.

Using a Bayesian approach and the Markov Chain Monte Carlo (MCMC) sampling method, a posterior distribution was generated for each coefficient of the model in (1). The average of the sampled values was used as an estimate for the coefficient, and 95% credibility intervals were used as a criterion to determine whether the covariates should remain into the model. As a prior distribution to the intercept and the covariate coefficients, it was adopted a flat distribution and a normal distribution with a zero mean and a variance equal to 1.0×10^−5^, respectively. For the precision parameters σ^2^_μ_ and σ^2^_s_, we adopted a gamma distribution (0.5; 0.0005). The simulations were made using the software WinBugs 1.4. Details pertaining to spatial statistical modeling for the relative risk of VL are available in supporting information.

## Results

Geocoding was possible for 93% of the infected dogs (34,127/36,627) and 93% of the human VL cases (384/412). The remaining cases were excluded due to inconsistencies in the addresses. Among the 28 cases of human VL whose residential address was not geocoded (7%), 11 were homeless and 17 could be allocated to a sanitary district, but not in the geographic units of analysis (146 small-areas). The loss on geocoding can be considered homogeneous in all health districts of Belo Horizonte. This allows us to consider that the lack of geocoding of cases is not associated with the occurrence of VL or other variables.

**Figure 2:**
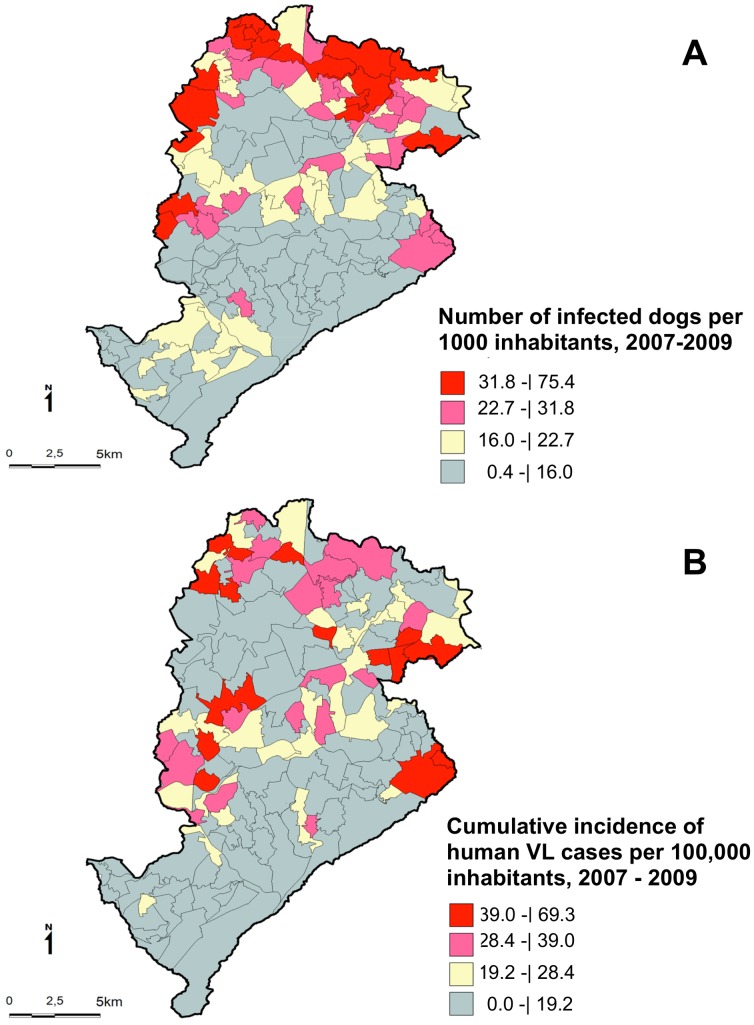
Spatial distribution of the number of infected dogs per 1,000 inhabitants (A) and the cumulative incidence rates of the human visceral leishmaniasis cases per 100,000 inhabitans (B), Belo Horizonte (Brazil), 2007–2009. The categories of both maps were defined using the quartiles.

The Figure 2 shows the spatial distribution of the number of infected dogs per 1,000 inhabitants (A) and the cumulative incidence rates of the human visceral leishmaniasis cases per 100,000 inhabitants (B). Because of the missing cases, this figure underestimates the number of infected dogs and human VL cases. In Figure 2-A, the higher levels of canine infection are concentrated in five of the nine health districts of the city. In Figure 2-B, the highest cumulative incidence rates of human VL (crude rates) are located in six health districts. These maps show the overlap of human VL cases and infected dogs.

**Figure 3:**
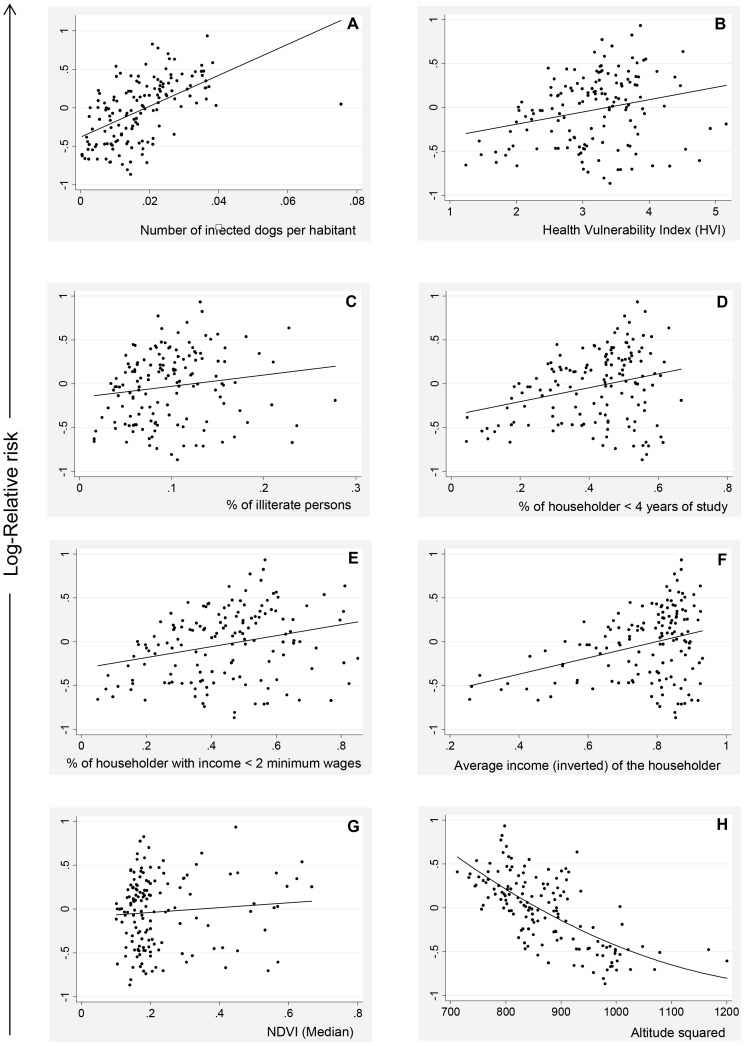
Relation between the log-relative risk of human visceral leishmaniasis (Y axis) and the covariates included in the spatial analysis (X axis), Belo Horizonte (Brazil), 2007–2009. The points represent each of the 146 spatial units of analysis. A) number of infected dogs to inhabitants; B) Health Vulnerability Index; C) percentage of illiterate people; D) percentage of householder <4 years of education; E) percentage of householder with income <twice the Brazilian minimum wage; F) average income (inverted) of the householder; G) Normalized Difference Vegetation Index-NDVI; H) altitude.

The scatter plots in Figure 3 present the log-RR-VL on the vertical axis and the covariates on the horizontal axis. The points represent each of the 146 coverage areas. We observed a positive linear trend between the log-RR-VL and the canine infection represented by the covariate “number of infected dogs to inhabitants” (Figure 3-A). The graphs for Health Vulnerability Index (Figure 3-B) and its components that displayed a positive linear trend with log-RR-VL are also presented (Figure 3-C to 3-F), as follows: percentage of illiterate people; percentage of householder with fewer than four years of education; percentage of householder with an income fewer than twice the Brazilian minimum wage (US$ 200.00) and average income (inverted) of the householder. Since a smaller income usually results in a higher vulnerability of the population of an area, the inverted average income was used in the analysis to preserve a possible positive relation with the relative risk. All these covariates were selected for further statistical modeling of the log-RR-VL.

The following Health Vulnerability Index components did not display evidence of an association with the occurrence of VL: proportion of the householders aged 10–19 years and indicators of inadequate or absent services of water supply, sanitary sewage and waste disposal.

It was observed lack of association between the log-RR-VL and the NDVI (Figure 3-G). However, the NDVI was included in the modeling spatial because of the vegetation coverage is favorable to the vector presence.

The locations of the residence of the human VL cases exhibited between 716 and 1,143 meters of the altitude. A non-linear relation between the log-RR-VL and altitude was found (Figure 3-H). In general, a lower altitude indicated a higher log-RR-VL in BH, with a higher frequency of cases below 949 meters.

Table 1 shows the results of the estimates parameters of the univariate spatial models for the log-relative risk of VL. Except for the NDVI and altitude, the other covariates displayed coefficients whose means may be considered to be significantly different from zero, since their 95% credibility intervals do not include the value of zero.

**Table 1:** Estimates parameters of the univariate spatial models for the log-relative risk of visceral leishmaniasis, Belo Horizonte (Brazil), 2007–2009.

Variable	Coefficient (mean)	95% CI(a)
Number of infected dogs per inhabitant	19.11	(6.65; 31.14)
Health vulnerability index (IVS)	0.312	(0.12; 0.49)
% mean of illiterate persons	3.211	(0.24; 6.07)
% mean of householder with less than 4 years of education	1.824	(0.81; 2.80)
% mean of householder with less than 2 minimum wages	1.183	(0.42; 1.92)
Average income (inverted) of the householder	2.014	(1.05; 2.96)
NDVI	0.01631	(−1.22; 1.22)
Altitude squared	−0.00109	(−0.01439; 0.01498)

(a) 95% credibility interval.

The full models with the best fit to the data to explain the relative risk of VL in Belo Horizonte comprised two variables: number of infected dogs per inhabitants and education or income (models 1 to 4). Model 1 presented the lowest deviance information criterion (DIC) value and, therefore, fit slightly better to the data (Table 2). According to this model, an increase of 0.01 infected dogs per inhabitant leads to an average increase of 13% in the relative risk of VL in areas with the same mean householder income. Considering areas that have the same number of infected dogs per inhabitants, a decrease of 10% in the mean householder income, which means an increase of 11.1% in the inverted mean income, makes the relative risk of VL to be multiplied by 6.6, on average.

**Table 2:** Estimates parameters (smoothed mean) of the full spatial models for the log-relative risk of visceral leishmaniasis, Belo Horizonte, Brazil, 2007–2009.

Model	Log-relative risk		DIC(b)
	Mean	95% CI(a)	
**Model 1**			
Number of infected dogs/inhabitant	12.19	0.17–23.77	538.300
Income (inverted) of the householder	1.70	0.80–2.66	
**Model 2**			
Number of infected dogs/inhabitant	18.62	6.65–29.77	545.270
% mean of illiterate persons	2.87	0.05–5.54	
**Model 3**			
Number of infected dogs/inhabitant	13.19	0.52–25.13	539.011
% mean of householder with fewer than 4 years of education	1.52	0.44–2.58	
**Model 4**			
Number of infected dogs/inhabitant	15.92	4.55–26.87	540.615
% mean of householder with fewer than twice the minimum wage	1.02	0.23–1.78	

(a) 95% credibility interval.

(b) deviance information criterion.

**Figure 4:**
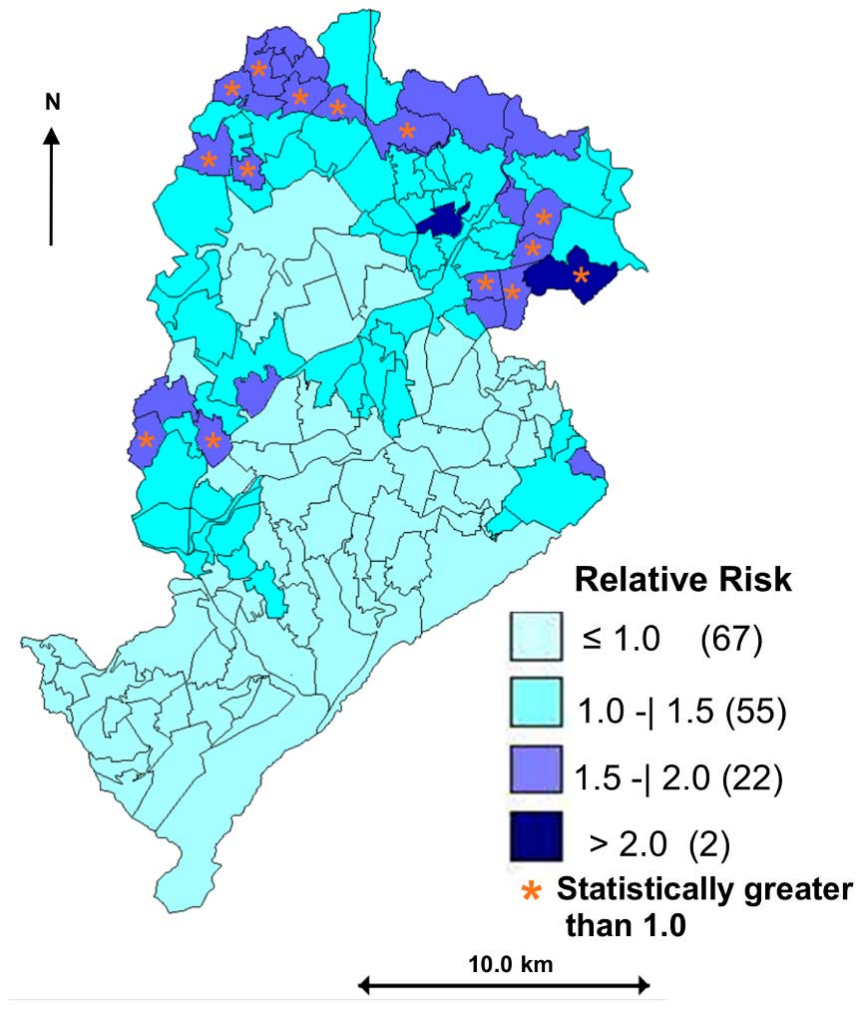
Spatial distribution of the smoothed estimates (mean) for the relative risk of visceral leishmaniasis (model 1), Belo Horizonte (Brazil), 2007–2009. The asterisks indicate the coverage areas whose relative risk is considered statistically greater than 1.0 (95% credibility interval).

The map of this model highlights the 14 areas with the highest RR-VL in Belo Horizonte (Figure 4). The estimates of RR-VL ranged from 0.42 to 2.55 and in 54% of the areas (79/146) they were greater than 1.0. The estimates of RR-VL are significantly higher than 1.0 (inferior limit of the 95% credibility interval) in 14 areas and 12 of them are concentrated in the northern region of the city.

## Discussion

In this study, the relative risk of human visceral leishmaniasis was shown to be correlated with income, education and the number of infected dogs per inhabitants in Belo Horizonte.

The indicators of income and education of the Health Vulnerability Index were significantly associated with the relative risk of VL, which suggests the usefulness of this index in the planning and prioritization of areas for control actions in Belo Horizonte. It should be stressed that income and education are related to each other and to several health problems. Therefore, income can serve as a proxy for the socio-economic-cultural context. Although the ecological correlations do not allow for causal inferences [28], this study has permitted a joint analysis of the intra-urban differences that are potentially associated with the occurrence of human VL cases.

In Belo Horizonte, other studies suggest intra-urban differences related to risk of VL. These studies suggest high indices of vector diseases in the poorest regions of the city [29], and a low socioeconomic level of dog owners and canine infection by *L. infantum* [19].

Ecological studies performed in Teresina (the capital of Piauí State, northeastern Brazil) suggest a spatial correlation between a high incidence of VL and areas with less urban infrastructure and precarious life conditions [6], [30]–[31]. In Belo Horizonte, the wide distribution of water supply (98.7% of the population), sewage (91.1%), and waste collection (97.9%) services [20] likely reflect the higher degree of urbanization of the city.

In Belo Horizonte, the ratio between the number of infected dogs per inhabitants in geographic areas and the occurrence of the human VL cases in these areas has not been described in the literature. The use of this indicator was only possible due to the large number of tested dogs (>150,000 dogs/year), which was equivalent to more than half the dog population, and to the existence of an information system to record control activities of the canine reservoir.

In Iran, an increase in seropositivity in children was associated with an increase in the canine population and the dog/human ratio [32]. However, canine infections were not evaluated, which is the main methodological difference with the present study. In Belo Horizonte, a spatial correlation between human cases and canine infections was suggested [13] in addition to a greater likelihood of human cases due to the presence of animals in the neighboring area [4]. Study conducted in Araçatuba (São Paulo State, southeastern region), showed that a higher concentrations of human cases were detected in areas with a higher prevalence of infected dogs [33]. Ecological association between canine infections and human VL cases were influenced by the worse socioeconomic conditions in a study carried out in Teresina [30]. All these results highlight the need for new control strategies aimed at infected dogs in urban areas.

As pointed out in our results, in the multivariate spatial analysis, the altitude did not remain in the final-models. Nevertheless, another study conducted in Belo Horizonte suggested a concentration of infected dogs and human VL cases between 780 and 880 m [14]. In Sudan, the average rainfall and the altitude were the best predictors of VL incidence [34]. In addition, in India, altitude was associated to the risk of VL, and poverty was cited as a determinant of transmission of the disease [35].

Vegetation, represented by the NDVI, was not shown to be associated with the relative risk of VL in Belo Horizonte. Elsewhere, it was identified as an environmental factor predictive of disease risk [30]–[31], [34]–[36]. In Teresina, higher VL incidence rates in areas with worse socioeconomic conditions, high population growth, and abundant vegetation suggested a relation between the occupation of the area and its vegetation coverage, which was represented by the NDVI [30]. It is probable that the differences in levels of urbanization and infrastructure of Teresina and Belo Horizonte have leaded to these different results.

In Brazil, VL is a disease requiring mandatory reporting and whose drugs for treatment are provided exclusively by the government and released only after reporting the case to the Brazilian Reportable Disease Information System. In addition, a three-decades of the occurrence of the disease in Belo Horizonte, the capacity to care for cases through the healthcare network, and the existence of an organized health surveillance system allow for the assumption that there is very little underreporting of cases. However, because of the missing geocoding (7%), the estimates in Figure 2 include an underestimated number of cases.

The use of spatial statistical modeling allowed for estimations of incidence rates smoothed by the spatial dependence between neighboring areas. This Bayesian approach minimizes the instability of rates resulting from a low frequency of cases in small-areas, eliminates a large part of the randomness not associated with the risk factors, and overcomes the political-administrative delimitations of the areas [6], [23]–[24], [26]–[27]. Therefore, this approach proved to be useful for the identification of small-areas with a higher risk of VL and exhibited its operational applicability in surveillance and control in an urban environment with an unequal spatial distribution of the disease.

Some limitations of this study should also be highlighted. One limitation is related to the temporality of the Health Vulnerability Index, which uses socioeconomic indicators taken from the demographic census of the year 2000 [20]. The updating of this index depends on the results of the census from 2010, which was no available during this study.

The broad distribution of the vector *L. longipalpis* has been described in Belo Horizonte and, more precisely, in the peridomiciles of the households [12], [14]–[15]. Based on this knowledge, households were considered to be likely infection sites. Therefore, the geocoding of human VL cases in the level of the household can be a limitation of this study because there would be an ideal indicator of the risk of transmission for all situations.

In spite of the fact that Belo Horizonte has high coverage of geocoding system, some problems related to the absences or inconsistencies in addresses could be happen at the time of human VL case reporting and during the canine survey (eg. homeless individuals; house number inexistent). Loss in geocoding of human cases and canine infection was around 7% and it can be considered homogeneously distributed over the city. Hence, it is likely that the loss was not associated with geographical locations and suggested absence of selection bias in spatial analysis [37].

Areas of the municipalities neighboring to Belo Horizonte were not included in the Bayesian analysis to estimate incidence rates. This point would be other limitation of our study. It is noteworthy that georeferenced data of VL patients from neighboring counties may not be available due to the lack of the geocoding system.

In Belo Horizonte, it is likely that the effectiveness and sustainability of the visceral leishmaniasis control program are influenced by the complexity of the disease in a large territorial area with high human and canine population densities and intra-urban differences. In this context, identifying higher risk areas may help in the surveillance of VL, direct the prioritization of small-areas for specific interventions [30], [38], and contribute to the effectiveness and reduction of operational costs of the Visceral Leishmaniasis Control and Surveillance Program [6], [39]–[40].

## Supporting Information

Model S1 Spatial statistical modeling: script and results of the models S1.
(DOC)
