# Supplementary material for: Relative Risk of Visceral Leishmaniasis in Brazil: A Spatial Analysis in Urban Area
Source: PLoS Negl Trop Dis. 2013 Nov 7;7(11):e2540. doi: 10.1371/journal.pntd.0002540 (PMC3820760; doi:10.1371/journal.pntd.0002540)

**Supporting Information**

**Spatial statistical modeling: script and results of the models S1**

**Script S1**

**Script of Spatial statistical modeling for the relative risks of VL**

**(performed using the WinBUGS 1.4 software)**

model {

# Likelihood

for (i in 1 : N) {

O[i] ~ dpois(mu[i])

log(mu[i]) <- log(E[i]) + alpha0 + alpha1*caopop[i] + alpha2*ivs[i] + alpha3*alfabivs[i] + alpha4*chefe4ivs[i] +

alpha5*chefe2ivs[i] + alpha6*rendainvivs[i]+ alpha7*ndvi[i] + alpha8*(alt2[i]/1000) + b[i]

b[i] <- mu[i] + s[i]

RR[i] <- exp(alpha0 + alpha1*caopop[i] + alpha2*ivs[i] + alpha3*alfabivs[i] + alpha4*chefe4ivs[i] +

alpha5*chefe2ivs[i] + alpha6*rendainvivs[i]+ alpha7*ndvi[i]+ alpha8*(alt2[i]/10000) + b[i])

}

# Mu prior distribution for non-structured random effects:

for(k in 1:N) {

mu[k] ~ dnorm(0.0, tauMu) }

# CAR prior distribution for random effects:

s[1:N] ~ car.normal(adj[], weights[], num[], tauS)

for(k in 1:sumNumNeigh) { weights[k] <- 1 }

# Other priors:

alpha0 ~ dflat()

alpha1 ~ dnorm(0.0, 1.0E-5)

alpha2 ~ dnorm(0.0, 1.0E-5)

alpha3 ~ dnorm(0.0, 1.0E-5)

alpha4 ~ dnorm(0.0, 1.0E-5)

alpha5 ~ dnorm(0.0, 1.0E-5)

alpha6 ~ dnorm(0.0, 1.0E-5)

alpha7 ~ dnorm(0.0, 1.0E-5)

alpha8 ~ dnorm(0.0, 1.0E-5)

tauS ~ dgamma(0.5, 0.0005) # prior on precision of s

sigmaS <- sqrt(1 / tauS) # standard deviation of s

tauMu ~ dgamma(0.5, 0.0005) # prior on precision of mu

sigmaMu <- sqrt(1 / tauMu) # standard deviation of mu

}

Data

list(N = 146, O = c(2,1,2,0,2,0,2,0,1,3,0,0,1,2,1,2,1,0,3,5,8,9,5,4,2,2,7,3,4,2,2,1,2,2,7,6,6,3,4,6,5,0,0,6,5,1,2,3,6,5,7,7,1,4,4,4,0,7,2,2,1,2,3,3,4,2,3,3,3,8,7,3,1,0,0,2,4,1,2,1,0,4,2,1,1,4,4,3,4,0,0,0,0,0,4,4,5,1,3,1,0,0,1,1,0,2,2,1,1,1,1,0,7,1,3,2,3,5,1,7,6,5,0,2,0,8,1,2,0,1,2,1,2,2,4,4,4,3,1,1,2,1,2,5,6,2),

**E** = c(2.451539807,2.500090246,1.616918312,1.567510092,2.169501218,2.130386377,2.784530624,

2.467494545,2.59684801,3.220798344,1.245155768,1.563564297,2.634075731,2.080463488,

2.855211827,1.554814925,2.488595972,3.394756453,2.859672292,1.938071743,2.137934855,

2.370222112,4.351182934,1.926405913,1.371078106,1.962604296,3.076004811,2.640423315,

2.513986308,2.288904416,0.922801443,5.320990809,2.456343384,1.315665415,4.123699255,

2.687086634,2.763086084,2.778011484,2.395612447,5.13262197,2.083894614,0.541260188,

1.335222836,3.546412239,3.242414441,2.885234183,3.749363365,2.599249799,2.842345103,

3.875628816,2.413625861,3.442620666,1.609884503,2.417056987,1.376396352,2.14188065,

1.60679649,3.528055713,2.874769248,3.507468955,7.570608828,4.228863279,1.627897917,

2.454113152,6.318247695,1.620864108,4.2362402,2.098133789,2.641624209,3.325790812,

2.87048034,4.325621043,1.65946428,1.628755698,1.747472672,1.749702904,1.789160857,

1.467835871,1.789160857,1.733748166,0.633729045,2.619150332,2.986623968,7.749370513,

2.302285809,7.547963394,2.012698742,6.545731375,3.518791672,4.717112564,2.695836006,

0.730658365,1.44038686,2.257166497,5.523941935,3.811981422,2.086639515,2.322529455,

2.984050623,3.004808938,1.207070265,1.277923025,1.758452276,2.133645947,1.798081786,

1.437470402,3.275867923,3.079092824,1.802885363,1.636647289,2.566310985,1.962089627,

2.993314665,2.009610728,3.995032015,4.091103554,2.330935715,3.484480408,7.025059732,

6.088362227,5.740446011,2.133645947,0.376909234,1.848176231,0.919713429,2.860358517,

1.744556214,1.917656541,1.263340738,0.676789681,1.516557865,3.318585447,2.220453445,

2.527024588,0.990566189,2.612631191,1.991082645,1.501804022,1.511582732,2.28873286,

1.590841752,0.959686052,1.48464839,2.735980185,3.290964879,1.374337676),

**caopop** = c(0.0147655703289013, 0.0144788307143347,

0.0178249336870027, 0.00229834737878954, 0.0208761663767199,

0.00740860041874698, 0.0137391411496519, 0.0086212890217618,

0.0178370879302372, 0.00916160647704272, 0.000413337007440066,

0.010752688172043, 0.0118535886413964, 0.0173991918858745,

0.0210298624046146, 0.0154474235904226, 0.00703157314214808,

0.0133919547200323, 0.0150578918951347, 0.0256705319996459,

0.0223880597014925, 0.0207730167921251, 0.00500729408981587,

0.0247573247840413, 0.0279029029029029, 0.0318181818181818,

0.0253764640267708, 0.0185173153141446, 0.0227241708748465,

0.0145405486433818, 0.0198921732664064, 0.00880190869228785,

0.0224891744657075, 0.0262094145260138, 0.0172234471855889,

0.0227925684734725, 0.0264497702719483, 0.00512567158648799,

0.0254941277570897, 0.00972658600173808, 0.0115254795422738,

0.00507131537242472, 0.00925093151740974, 0.035265092879257,

0.0238095238095238, 0.00808657390890712, 0.0221002059025395,

0.0132004488152597, 0.0103814582327378, 0.037182948961976,

0.0293553202075485, 0.0169432401455125, 0.016304347826087,

0.0139115622116545, 0.0145830736632182, 0.0239487384861834,

0.00747384155455904, 0.00544614636518356, 0.00549024288357104,

0.0131572511616532, 0.00484941874957511, 0.00880324543610548,

0.00959005163873959, 0.00838867528836071, 0.00339406446007223,

0.00275190516511431, 0.015429473939983, 0.0212591986917416,

0.0223405637095727, 0.0272361497988239, 0.0382500597657184,

0.0231220750376775, 0.0180915951617906, 0.0191700021065936,

0.0392695857058708, 0.0247083047357584, 0.032313740531211,

0.0105189340813464, 0.0232045258414038, 0.0229566594102513,

0.0162425554953979, 0.0165716905744416, 0.00695042793957149,

0.00121759535985477, 0.00931445603576751, 0.00247744164374844,

0.0109955676781452, 0.00345957279517757, 0.00175515577007459,

0.000472796043060809, 0.0021636757031946, 0.00727870392110824,

0.0132205812291567, 0.00858858402371361, 0.0113978694990528,

0.0178217821782178, 0.0222806873304283, 0.00428423696262373,

0.00201218811084282, 0.00770767913217242, 0.0117964752700398,

0.00966572694321385, 0.0271219512195122, 0.019458068666077,

0.00457971567598512, 0.0159923618570235, 0.0169154228855721,

0.011199019389347, 0.00846893139214007, 0.0120545073375262,

0.0135704258306037, 0.0144268601906094, 0.0357060981201284,

0.0192077855557453, 0.021084725383261, 0.0146349645657735,

0.00250239199234562, 0.00694205110531239, 0.0102078194827713,

0.016202203499676, 0.0148531125788231, 0.0291066977566937,

0.0227583067819754, 0.0181936322287199, 0.0335758254057079,

0.0188928207281233, 0.0316648638017504, 0.0272857398461263,

0.0186040195545899, 0.0167300380228137, 0.0102941176470588,

0.0134408602150538, 0.0363130649772078, 0.0114052953156823,

0.0367163145133356, 0.0349333508437849, 0.0350680682405652,

0.0360977838702307, 0.0308705027806151, 0.0754066411813207,

0.0364499083360293, 0.0362888809438684, 0.0249595562745551,

0.0332330072736393, 0.0261690038054527, 0.0214704780926226),

**ndvi** = c(0.168831, 0.236364, 0.195122,

0.417636, 0.185185, 0.215535, 0.179638, 0.540984, 0.304348, 0.2,

0.569231, 0.167749, 0.175, 0.175, 0.211268, 0.153846, 0.147541,

0.153846, 0.190476, 0.348481, 0.170213, 0.180723, 0.116279, 0.333333,

0.297297, 0.2, 0.195122, 0.157895, 0.13253, 0.178947, 0.196262,

0.168831, 0.142857, 0.306667, 0.138462, 0.148239, 0.135135, 0.157895,

0.212766, 0.164835, 0.142857, 0.151515, 0.127273, 0.152174, 0.162162,

0.153846, 0.173333, 0.170213, 0.238517, 0.142857, 0.142857, 0.188406,

0.173333, 0.16129, 0.189873, 0.194576, 0.111111, 0.22807, 0.117265,

0.186441, 0.148936, 0.164179, 0.157072, 0.178082, 0.130435, 0.103963,

0.178947, 0.431699, 0.169231, 0.162162, 0.182796, 0.182796, 0.321101,

0.204819, 0.568627, 0.178571, 0.452632, 0.152174, 0.152941, 0.25,

0.623762, 0.157895, 0.180723, 0.170732, 0.2, 0.452055, 0.2, 0.16129,

0.232558, 0.104478, 0.128205, 0.489996, 0.333333, 0.285714, 0.210526,

0.186813, 0.315068, 0.291667, 0.408771, 0.223881, 0.195122, 0.146667,

0.205128, 0.384615, 0.166667, 0.367089, 0.164179, 0.175, 0.180328,

0.186813, 0.142857, 0.139785, 0.2, 0.159091, 0.134021, 0.125,

0.101124, 0.101449, 0.129032, 0.2, 0.165026, 0.557895, 0.531915,

0.270887, 0.5, 0.161598, 0.182796, 0.150184, 0.252174, 0.564787,

0.638889, 0.150685, 0.295238, 0.243902, 0.447154, 0.22449, 0.237113,

0.666667, 0.285714, 0.142857, 0.189873, 0.595745, 0.2, 0.173913,

0.172414, 0.149425),

**ivs** = c(2.97, 3.74, 3.68, 4.31, 3.03, 4.05, 3.06,

3.01, 3.24, 3.53, 4.76, 3.03, 2.87, 2.26, 3.3, 2.63, 3.83, 2.59,

3.13, 4.51, 3.29, 3.74, 2.03, 3.82, 3.8, 3.2, 3.44, 3.29, 2.62,

3.21, 2.67, 2.16, 3, 3.38, 2.52, 2.74, 2.82, 2.52, 3.68, 2.63,

2.48, 3.2, 3.93, 3.29, 3.58, 2.54, 3.4, 2.84, 3.27, 2.92, 2.76,

2.57, 3.37, 3.14, 3.28, 3.25, 3.4, 3.19, 3.72, 2.8, 2.01, 3.01,

3.54, 3.33, 1.91, 3.66, 3.08, 3.24, 3.5, 3.18, 3.33, 2.99, 3.8,

3.53, 3.69, 3.17, 3.69, 3.65, 3.01, 3.71, 4.39, 3.32, 2.31, 1.24,

2.94, 1.89, 3.79, 1.84, 1.44, 1.7, 1.47, 3.65, 2.14, 2, 2.24,

2.7, 3.08, 1.69, 3.71, 2.63, 3.15, 2.81, 2.88, 2.95, 4.55, 5.16,

2.77, 2.99, 3.57, 3.08, 3.32, 3.41, 3.28, 3.51, 2.39, 2.04, 2.72,

2.08, 1.95, 2.23, 2.53, 4.23, 4.92, 3.45, 3.47, 3.75, 3.34, 3.87,

3.9, 4.12, 3.92, 2.65, 3.84, 2.36, 3.87, 3.54, 3.95, 3.82, 3.59,

2.95, 3.5, 3.79, 3.19, 3.26, 2.88, 4.47),

**alfabivs** = c(0.068494179, 0.126600174,

0.138101451, 0.230837604, 0.058462555, 0.155328061, 0.082541676,

0.075941066, 0.078682424, 0.112433925, 0.168130292, 0.077490118,

0.07977103, 0.036916112, 0.116339212, 0.058095593, 0.174120285,

0.061699191, 0.095244068, 0.227319679, 0.08501692, 0.132901782,

0.033867973, 0.142295651, 0.126295644, 0.090943074, 0.116619735,

0.104051247, 0.049748586, 0.083446321, 0.059822701, 0.046557503,

0.066333937, 0.105064656, 0.057416075, 0.0629564, 0.062533708,

0.052650108, 0.119108895, 0.059945414, 0.058290102, 0.096141188,

0.147501241, 0.098149324, 0.125904156, 0.046953083, 0.107705086,

0.053933071, 0.09667682, 0.070124154, 0.060291467, 0.050164147,

0.07464363, 0.087456109, 0.090858546, 0.106965867, 0.128208007,

0.114955341, 0.152724026, 0.076116806, 0.051096513, 0.106543116,

0.120767864, 0.105538863, 0.030927596, 0.154647262, 0.08594761,

0.077074157, 0.124841986, 0.089148302, 0.106974533, 0.077014995,

0.135135099, 0.13417603, 0.108338057, 0.087735524, 0.122857957,

0.148142439, 0.086310853, 0.13288078, 0.195002786, 0.084604327,

0.084402552, 0.015892987, 0.081807387, 0.053680085, 0.156677391,

0.057956857, 0.024306935, 0.015788251, 0.017521769, 0.12374333,

0.05460993, 0.058037761, 0.039977281, 0.062635639, 0.101937239,

0.038856855, 0.182348458, 0.059960498, 0.097111507, 0.054954759,

0.064406115, 0.08190218, 0.2359065, 0.277584664, 0.049609274,

0.075506883, 0.112992989, 0.083777332, 0.099785846, 0.106188709,

0.0887384, 0.112868585, 0.043288052, 0.038525831, 0.071709691,

0.03975095, 0.038204419, 0.041886339, 0.067508233, 0.169078182,

0.208622915, 0.105898042, 0.0938657, 0.149835265, 0.102173544,

0.122662098, 0.156225856, 0.158031293, 0.180791167, 0.064066461,

0.131206137, 0.064671905, 0.130968593, 0.119088624, 0.136924497,

0.157485441, 0.103258237, 0.074526472, 0.103009033, 0.150357644,

0.089910907, 0.092967678, 0.082346436, 0.210248162),

**chefe4ivs** = c(0.45355623, 0.555533096, 0.532523628,

0.614145703, 0.378329528, 0.56486996, 0.454593164, 0.439383237,

0.450872759, 0.515518506, 0.607516612, 0.454619819, 0.432121597,

0.287676811, 0.510568621, 0.435985851, 0.560102482, 0.350400819,

0.451519427, 0.630676085, 0.502782086, 0.562725038, 0.196681582,

0.527158831, 0.521466758, 0.485934158, 0.516385305, 0.496447921,

0.38847057, 0.465448794, 0.318489322, 0.213116197, 0.463796159,

0.463059738, 0.359563079, 0.289334394, 0.448787446, 0.28075258,

0.523192012, 0.297837379, 0.306864121, 0.60655985, 0.559096284,

0.514991289, 0.495361013, 0.309957801, 0.463882208, 0.314903892,

0.381345418, 0.453326569, 0.414579418, 0.315453848, 0.45065598,

0.464091878, 0.501702209, 0.44703241, 0.540506519, 0.391485998,

0.52425396, 0.36063765, 0.170251475, 0.310232481, 0.496792281,

0.49394286, 0.162762036, 0.536025631, 0.355263117, 0.429175364,

0.545063946, 0.482331414, 0.474625104, 0.39399873, 0.530827528,

0.528688955, 0.506505154, 0.33367165, 0.470618139, 0.554507475,

0.450248901, 0.516354905, 0.577524767, 0.561968284, 0.224073508,

0.044516397, 0.295279197, 0.136973404, 0.413660319, 0.126415183,

0.047054784, 0.106684353, 0.089436755, 0.448015542, 0.205376201,

0.175088545, 0.182914701, 0.302837883, 0.291734101, 0.104702771,

0.417962849, 0.174242925, 0.446673337, 0.294356051, 0.373455705,

0.32005501, 0.596684624, 0.666121059, 0.383879488, 0.411207146,

0.571806899, 0.4760097, 0.566208341, 0.55351997, 0.452942622,

0.504959922, 0.344972662, 0.228837725, 0.36338203, 0.247390084,

0.181630114, 0.26365584, 0.295511021, 0.533850296, 0.542982076,

0.446115579, 0.380034369, 0.51108562, 0.34466916, 0.590146744,

0.583152051, 0.552579879, 0.580808673, 0.258159483, 0.465339131,

0.221983813, 0.540106863, 0.506653256, 0.55677384, 0.519959971,

0.448337761, 0.443318971, 0.488066064, 0.549952999, 0.404750676,

0.509202582, 0.415918434, 0.617298107),

**chefe2ivs** =c(0.38608525, 0.573338405, 0.60191171,

0.768700481, 0.309722764, 0.671460809, 0.37318881, 0.374306169,

0.387102317, 0.505771009, 0.632930931, 0.467576622, 0.402386551,

0.200499821, 0.48793122, 0.382255059, 0.709981249, 0.338120239,

0.461753971, 0.811717805, 0.519634274, 0.560067645, 0.172978377,

0.606243464, 0.598875136, 0.47732384, 0.553351233, 0.514805311,

0.34222474, 0.454189302, 0.286949506, 0.194551296, 0.465229162,

0.440171435, 0.334494555, 0.311495568, 0.428333921, 0.243546647,

0.566434815, 0.284136445, 0.328654844, 0.496602975, 0.723044791,

0.501986284, 0.523673219, 0.26447217, 0.505067533, 0.282975098,

0.375965824, 0.443182515, 0.392604976, 0.275626277, 0.416549354,

0.497171161, 0.537807173, 0.42335781, 0.620981504, 0.44462594,

0.622224968, 0.357513884, 0.196420402, 0.407341806, 0.473224791,

0.590232629, 0.143900463, 0.672762357, 0.376813962, 0.383514415,

0.58526839, 0.45522573, 0.506512101, 0.35435794, 0.575088207,

0.562986959, 0.502936182, 0.365575669, 0.555596681, 0.629583306,

0.482636362, 0.535494185, 0.807271862, 0.466397863, 0.270170091,

0.050394596, 0.350426105, 0.157659197, 0.578440166, 0.152197747,

0.082006168, 0.096700032, 0.07579126, 0.465023353, 0.207887641,

0.161471935, 0.176009938, 0.29747363, 0.308780685, 0.113111011,

0.552968719, 0.256307627, 0.441706299, 0.274829281, 0.365649606,

0.366835528, 0.824649292, 0.850803724, 0.27937859, 0.350634609,

0.535710459, 0.378932389, 0.472062986, 0.470853235, 0.464471111,

0.547812888, 0.307367188, 0.197294155, 0.353073575, 0.224631029,

0.157454202, 0.227158855, 0.289202122, 0.65489022, 0.808157492,

0.53128628, 0.411966723, 0.600400024, 0.380019001, 0.644028091,

0.653172186, 0.694584008, 0.747502966, 0.279003746, 0.550047459,

0.236951268, 0.565569731, 0.57309193, 0.591818399, 0.651419979,

0.497900962, 0.401117274, 0.488043161, 0.62114546, 0.437009599,

0.489640121, 0.390001339, 0.796531107),

**rendainvivs** = c(0.813007012, 0.880666368, 0.881604106,

0.919102755, 0.784343736, 0.900234354, 0.807255801, 0.800780312,

0.815753076, 0.850296689, 0.891885088, 0.826878574, 0.799635458,

0.6540062, 0.840568826, 0.787673997, 0.903209734, 0.717029673,

0.816849043, 0.924923595, 0.850764441, 0.869990695, 0.534028122,

0.880640578, 0.882962575, 0.834930683, 0.858278758, 0.836349879,

0.743360765, 0.814018094, 0.688969994, 0.526750917, 0.814704138,

0.830151259, 0.717588089, 0.673791342, 0.804643368, 0.588371504,

0.857527437, 0.664977325, 0.721890155, 0.844030702, 0.893212861,

0.846134777, 0.820082382, 0.689744308, 0.817493013, 0.726793695,

0.75721028, 0.816267709, 0.784870628, 0.687685247, 0.813334521,

0.839593429, 0.85327111, 0.803746877, 0.883691303, 0.815240572,

0.882079847, 0.739376595, 0.455334508, 0.688363946, 0.829796503,

0.869519057, 0.526657029, 0.89551878, 0.759885542, 0.811707612,

0.87398678, 0.827871958, 0.83677971, 0.761321749, 0.86938805,

0.870352555, 0.856621731, 0.744161477, 0.853098319, 0.872334821,

0.816695912, 0.856342088, 0.925449096, 0.841264343, 0.504141658,

0.256811088, 0.613116113, 0.366948126, 0.717955098, 0.34824486,

0.28652145, 0.491905535, 0.422692644, 0.80866045, 0.475880637,

0.43124123, 0.576266817, 0.72000845, 0.658486859, 0.262709785,

0.728051282, 0.568227151, 0.804281365, 0.744948781, 0.778668734,

0.669056045, 0.925215864, 0.931294707, 0.730602457, 0.784191959,

0.86070798, 0.807597252, 0.851761978, 0.853847836, 0.830233966,

0.855484164, 0.692663138, 0.596786294, 0.747458839, 0.650188266,

0.569373269, 0.635580176, 0.658950679, 0.880153137, 0.922565967,

0.84751653, 0.719863935, 0.862224152, 0.754870911, 0.88942652,

0.893569005, 0.907871759, 0.906476421, 0.623466585, 0.858068236,

0.603993715, 0.870338018, 0.870233727, 0.873595044, 0.879201161,

0.842490373, 0.808245983, 0.850762722, 0.887614824, 0.795751683,

0.840932422, 0.798814589, 0.919037167),

**alt2** = c(995206,

1052792, 953357, 981058, 997123, 986426, 978495, 1145084,

1004290, 998590, 1441919, 749956, 676880, 718458, 695692,

735306, 722242, 697005, 706776, 862855, 625088, 628259,

710193, 594331, 563487, 697559, 641000, 635231, 717986,

617971, 689091, 684813, 683102, 540015, 746909, 691436,

756644, 799848, 827784, 808546, 826446, 789610, 801025,

812047, 766922, 716844, 735487, 875628, 835758, 752556,

769920, 589239, 625764, 654533, 645403, 576441, 633249,

819308, 826645, 791989, 881272, 861298, 832005, 801025,

787078, 795423, 637928, 691445, 677329, 632214, 648025,

607261, 1014829, 1001782, 576650, 628717, 589420, 640667,

795307, 584349, 537927, 874838, 870293, 854040, 933319,

1365019, 918312, 951600, 935104, 768301, 735664, 706734,

700074, 723400, 656192, 693056, 785608, 1163282, 1098730,

901210, 850939, 797832, 810000, 966170, 1043462, 1021898,

903777, 984114, 967540, 918779, 954529, 959992, 695578,

671546, 786078, 795462, 758092, 771161, 705684, 718862,

793658, 1014149, 690037, 698060, 703965, 655336, 650203,

589056, 570834, 506804, 558564, 662628, 730661, 649987,

637520, 642730, 708327, 619441, 645717, 643698, 675578,

555957, 610249, 657930, 652864, 553164),

**num** = c(6, 3, 4, 3, 5, 7, 5, 6, 5, 4,

6, 3, 7, 6, 5, 3, 4, 5, 6, 1,

4, 6, 7, 6, 5, 5, 5, 11, 7, 5,

4, 5, 4, 4, 5, 6, 5, 5, 3, 8,

4, 8, 4, 3, 6, 4, 6, 5, 7, 5,

7, 7, 5, 5, 7, 6, 5, 4, 7, 7,

6, 5, 5, 5, 9, 7, 6, 5, 4, 4,

4, 6, 7, 3, 8, 7, 7, 5, 4, 7,

5, 7, 6, 8, 4, 7, 4, 4, 5, 6,

6, 6, 5, 5, 9, 4, 9, 7, 6, 5,

5, 4, 5, 8, 5, 4, 5, 6, 3, 4,

2, 3, 5, 4, 6, 6, 10, 5, 8, 6,

8, 7, 3, 4, 4, 5, 5, 6, 3, 3,

5, 6, 4, 6, 3, 4, 2, 7, 3, 7,

4, 7, 7, 7, 7, 5),

**adj** = c(

107, 9, 8, 7, 6, 5,

74, 8, 6,

108, 10, 6, 5,

73, 11, 9,

108, 107, 6, 3, 1,

74, 10, 8, 5, 3, 2, 1,

107, 82, 73, 9, 1,

73, 11, 9, 6, 2, 1,

73, 8, 7, 4, 1,

108, 74, 6, 3,

104, 99, 98, 73, 8, 4,

16, 15, 13,

120, 19, 18, 16, 15, 14, 12,

120, 32, 31, 26, 16, 13,

123, 19, 17, 13, 12,

14, 13, 12,

123, 122, 19, 15,

121, 120, 119, 19, 13,

122, 121, 18, 17, 15, 13,

122,

30, 28, 27, 26,

135, 131, 80, 30, 27, 24,

120, 119, 36, 35, 33, 32, 28,

131, 81, 80, 34, 25, 22,

146, 142, 80, 34, 24,

31, 28, 27, 21, 14,

135, 30, 26, 22, 21,

143, 132, 80, 57, 36, 32, 31, 30, 26, 23, 21,

117, 47, 43, 37, 36, 35, 33,

80, 28, 27, 22, 21,

32, 28, 26, 14,

120, 31, 28, 23, 14,

36, 35, 29, 23,

142, 81, 25, 24,

119, 117, 33, 29, 23,

132, 92, 33, 29, 28, 23,

117, 115, 47, 43, 29,

116, 51, 49, 42, 41,

97, 45, 40,

118, 116, 115, 97, 51, 47, 45, 39,

49, 48, 42, 38,

116, 66, 65, 63, 49, 48, 41, 38,

117, 115, 37, 29,

79, 50, 49,

97, 51, 50, 46, 40, 39,

97, 96, 50, 45,

115, 97, 92, 40, 37, 29,

66, 63, 49, 42, 41,

79, 51, 48, 44, 42, 41, 38,

79, 51, 46, 45, 44,

116, 79, 50, 49, 45, 40, 38,

95, 78, 77, 76, 72, 67, 56,

141, 140, 128, 76, 56,

143, 140, 76, 57, 55,

140, 134, 132, 78, 76, 57, 54,

138, 128, 77, 76, 53, 52,

143, 132, 55, 54, 28,

66, 64, 60, 59,

102, 101, 82, 66, 64, 63, 58,

103, 100, 66, 65, 64, 62, 58,

99, 98, 90, 89, 65, 62,

100, 99, 65, 61, 60,

82, 66, 59, 48, 42,

103, 101, 60, 59, 58,

118, 117, 116, 90, 66, 62, 61, 60, 42,

65, 63, 60, 59, 58, 48, 42,

145, 127, 126, 95, 72, 52,

144, 139, 133, 77, 71,

145, 114, 113, 70,

145, 144, 136, 69,

144, 77, 72, 68,

145, 144, 77, 71, 67, 52,

104, 82, 11, 9, 8, 7, 4,

10, 6, 2,

146, 143, 142, 141, 140, 138, 129, 128,

140, 78, 56, 55, 54, 53, 52,

139, 138, 72, 71, 68, 56, 52,

134, 95, 76, 55, 52,

51, 50, 49, 44,

146, 143, 30, 28, 25, 24, 22,

142, 131, 130, 34, 24,

107, 104, 102, 73, 63, 59, 7,

121, 119, 105, 91, 85, 84,

91, 90, 89, 88, 87, 86, 85, 83,

105, 86, 84, 83,

122, 106, 105, 98, 88, 85, 84,

98, 89, 88, 84,

98, 87, 86, 84,

98, 90, 87, 84, 61,

117, 91, 89, 84, 65, 61,

121, 119, 117, 90, 84, 83,

134, 132, 97, 94, 47, 36,

124, 97, 96, 95, 94,

134, 97, 95, 93, 92,

134, 127, 125, 124, 94, 93, 78, 67, 52,

124, 97, 93, 46,

96, 94, 93, 92, 47, 46, 45, 40, 39,

99, 89, 88, 87, 86, 61, 11,

104, 100, 98, 62, 61, 11,

104, 103, 99, 62, 60,

104, 103, 102, 64, 59,

104, 101, 82, 59,

104, 101, 100, 64, 60,

103, 102, 101, 100, 99, 82, 73, 11,

121, 106, 86, 85, 83,

122, 121, 105, 86,

108, 82, 7, 5, 1,

110, 109, 107, 10, 5, 3,

112, 110, 108,

112, 111, 109, 108,

112, 110,

111, 110, 109,

127, 126, 125, 114, 69,

145, 126, 113, 69,

118, 117, 47, 43, 40, 37,

118, 65, 51, 42, 40, 38,

119, 118, 115, 91, 90, 65, 43, 37, 35, 29,

117, 116, 115, 65, 40,

121, 120, 117, 91, 83, 35, 23, 18,

119, 32, 23, 18, 14, 13,

122, 119, 106, 105, 91, 83, 19, 18,

123, 121, 106, 86, 20, 19, 17,

122, 17, 15,

125, 96, 95, 93,

127, 124, 113, 95,

145, 127, 114, 113, 67,

126, 125, 113, 95, 67,

141, 138, 129, 75, 56, 53,

138, 128, 75,

142, 131, 81,

135, 130, 81, 24, 22,

134, 92, 57, 55, 36, 28,

144, 137, 136, 68,

132, 95, 94, 92, 78, 55,

131, 27, 22,

144, 137, 133, 70,

136, 133,

142, 139, 129, 128, 77, 75, 56,

138, 77, 68,

143, 141, 76, 75, 55, 54, 53,

140, 128, 75, 53,

146, 138, 130, 81, 75, 34, 25,

146, 140, 80, 75, 57, 54, 28,

145, 136, 133, 72, 71, 70, 68,

144, 126, 114, 72, 70, 69, 67,

143, 142, 80, 75, 25

),

sumNumNeigh = 784)

**Inits**

list(tau = 1, alpha0 = 0,

alpha1=0,alpha2=0,alpha3=0,

alpha4=0,alpha5=0,alpha6=0,

alpha7=0, alpha8=0,

b=c(0,0,0,0,0,0,0,0,0,0,

0,0,0,0,0,0,0,0,0,0,

0,0,0,0,0,0,0,0,0,0,

0,0,0,0,0,0,0,0,0,0,

0,0,0,0,0,0,0,0,0,0,

0,0,0,0,0,0,0,0,0,0,

0,0,0,0,0,0,0,0,0,0,

0,0,0,0,0,0,0,0,0,0,

0,0,0,0,0,0,0,0,0,0,

0,0,0,0,0,0,0,0,0,0,

0,0,0,0,0,0,0,0,0,0,

0,0,0,0,0,0,0,0,0,0,

0,0,0,0,0,0,0,0,0,0,

0,0,0,0,0,0,0,0,0,0,

0,0,0,0,0,0))

**Results of the Models S1**

**Results of Spatial Statistical Modeling for the Relative Risks of VL**

**(performed using the WinBUGS 1.4 software)**

**Univariate Spatial Models**

alpha 1 = number of infected dogs per inhabitant


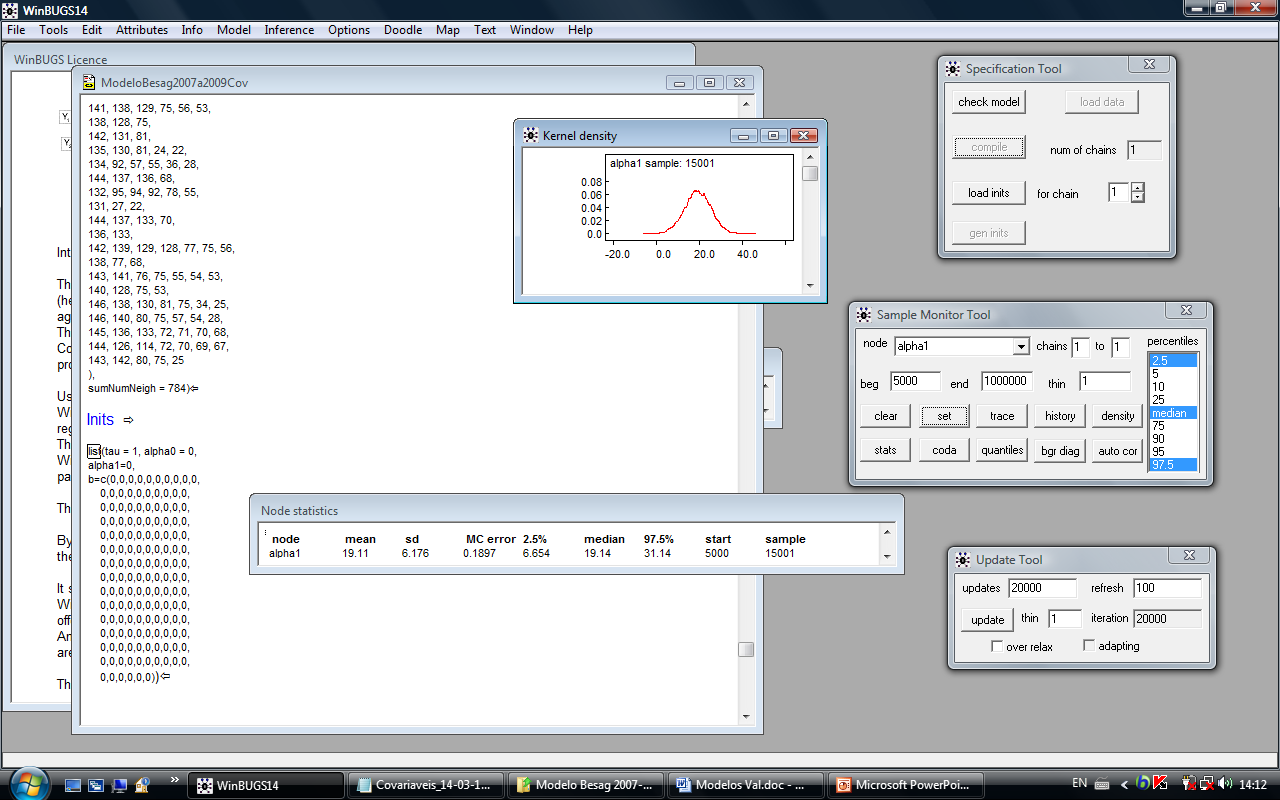


alpha 2 = average Health Vulnerability Index (Índice de Vulnerabilidade à Saúde-IVS)

**
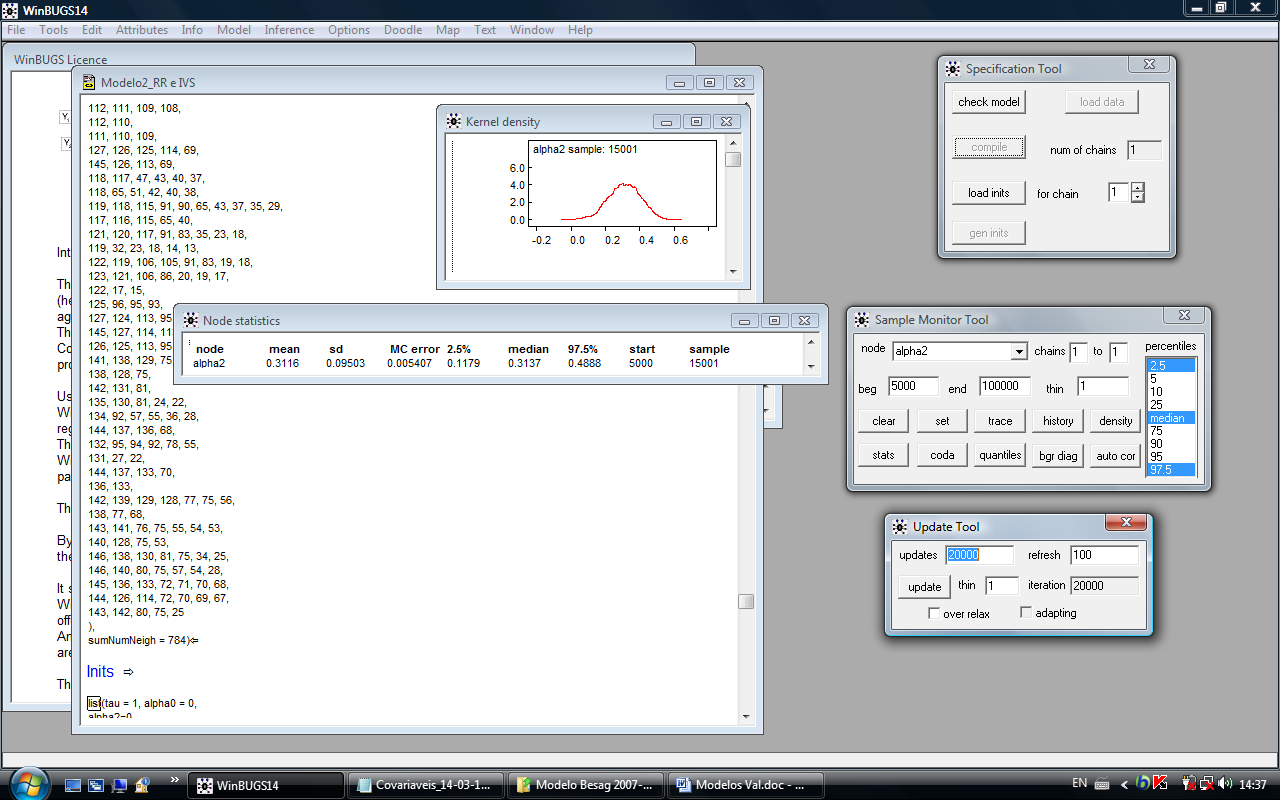
**

alpha 3 = percentage of illiterate people

**
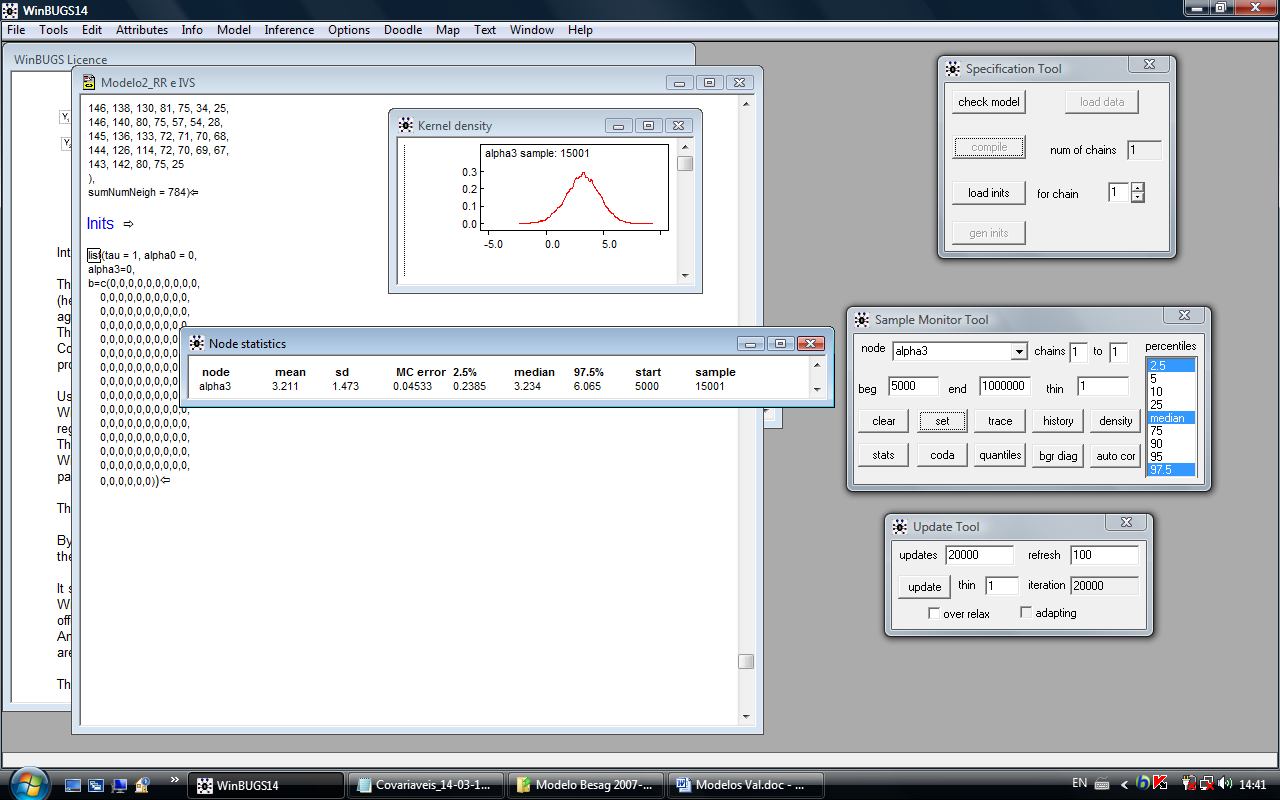
**

alpha 4 = percentage of householder with fewer than four years of education


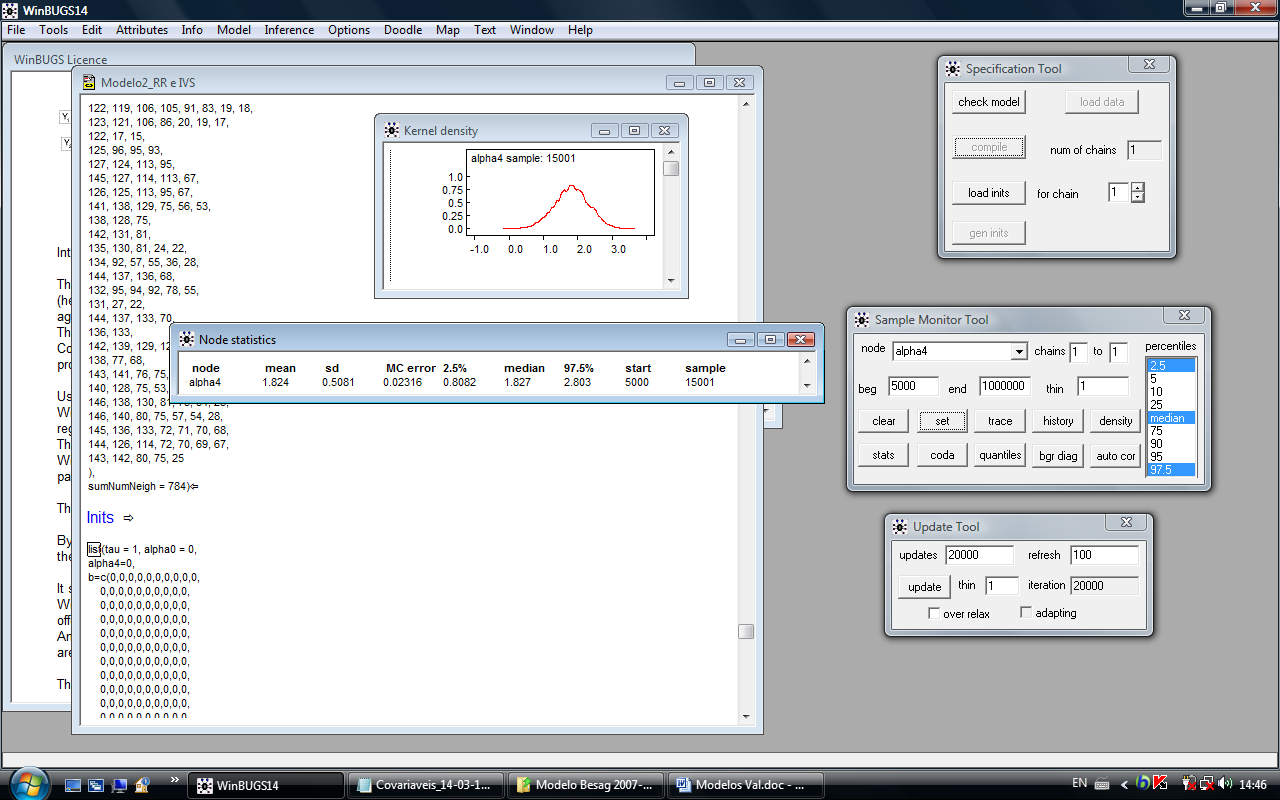


alpha 5 = percentage of householder with an income fewer than twice the Brazilian minimum wage (US$ 200.00)

**
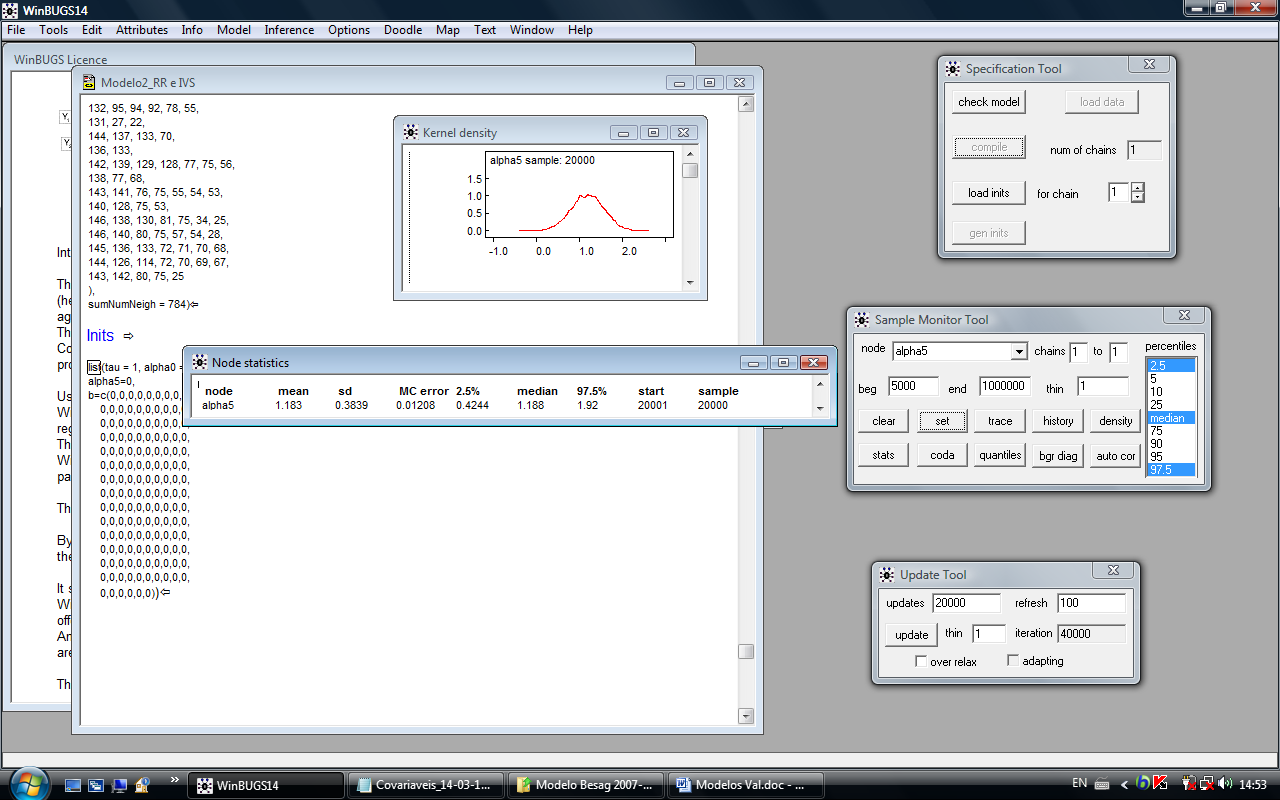
**

alpha 6 = average householder income (inverted)


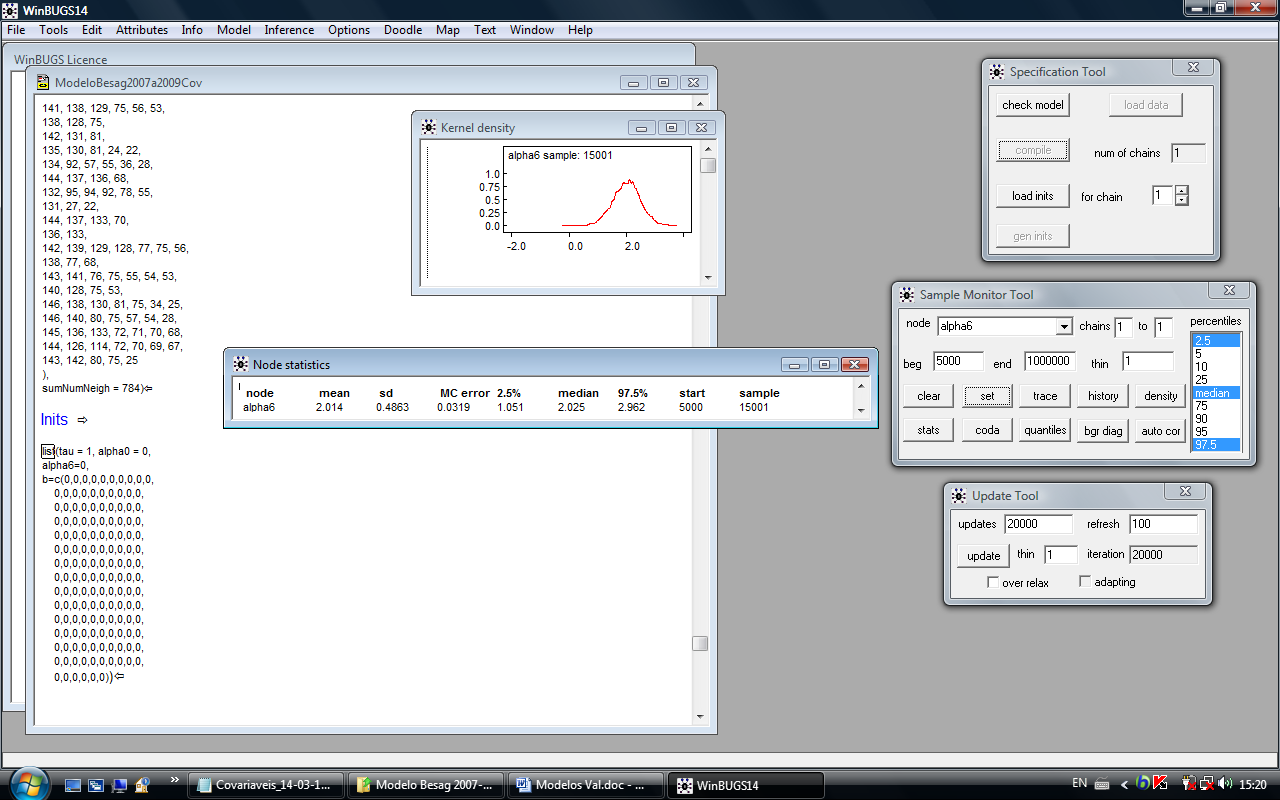


alpha 7 = NDVI (Normalized Difference Vegetation Index)


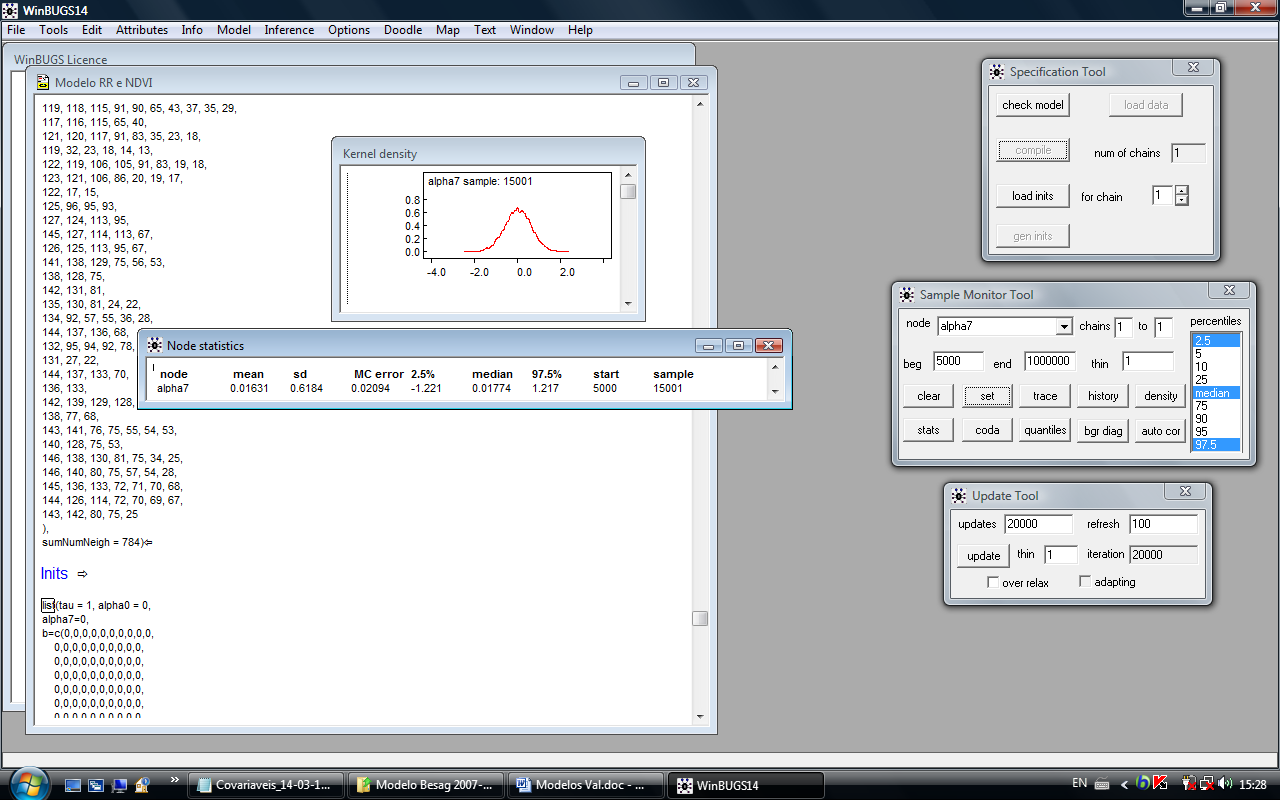


alpha 8 = altitude squared
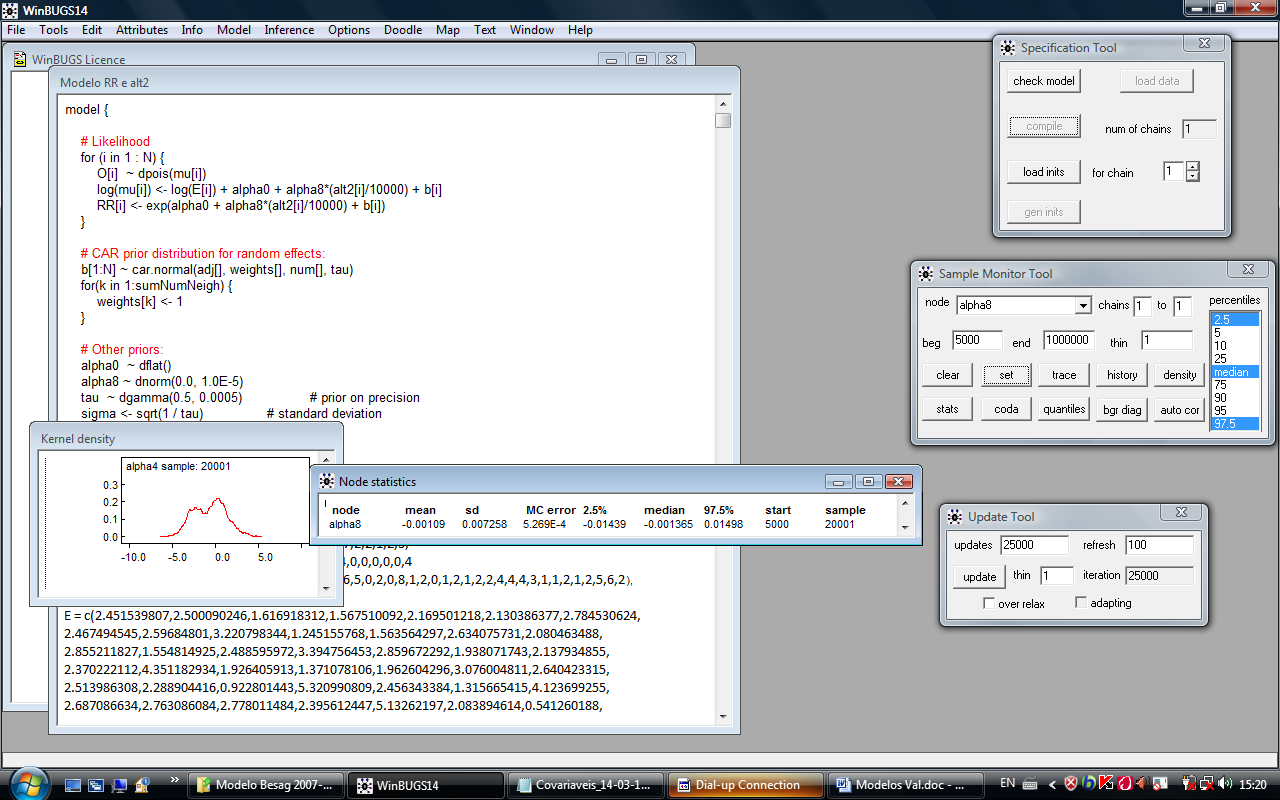


**Full Models**

**Model 1**

alpha1 and alpha6 = number of infected dogs per inhabitant and average householder income(inverted)
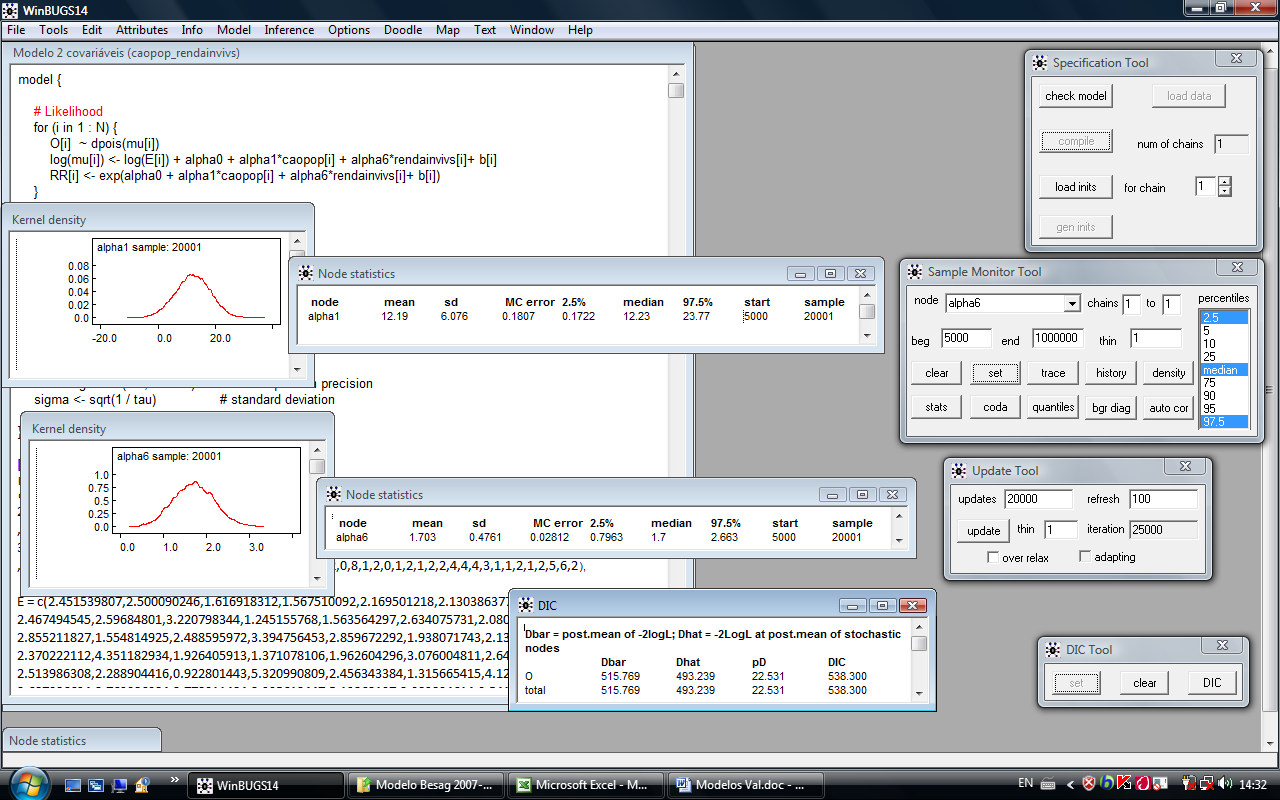


**Model 2**

alpha1 and alpha3 = number of infected dogs per inhabitant and percentage of illiterate people


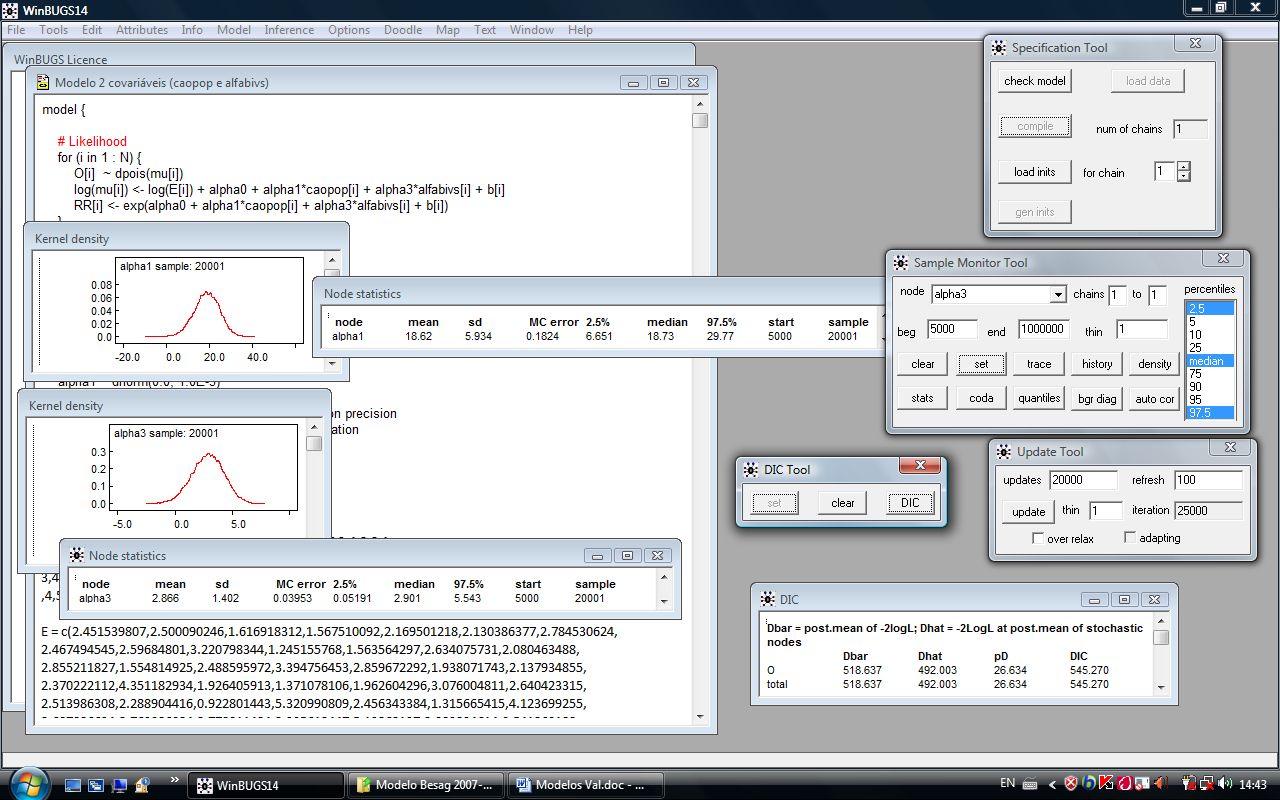


**Model 3**

alpha 1 and alpha 4 = number of infected dogs per inhabitant and percentage of householder with fewer than four years of education


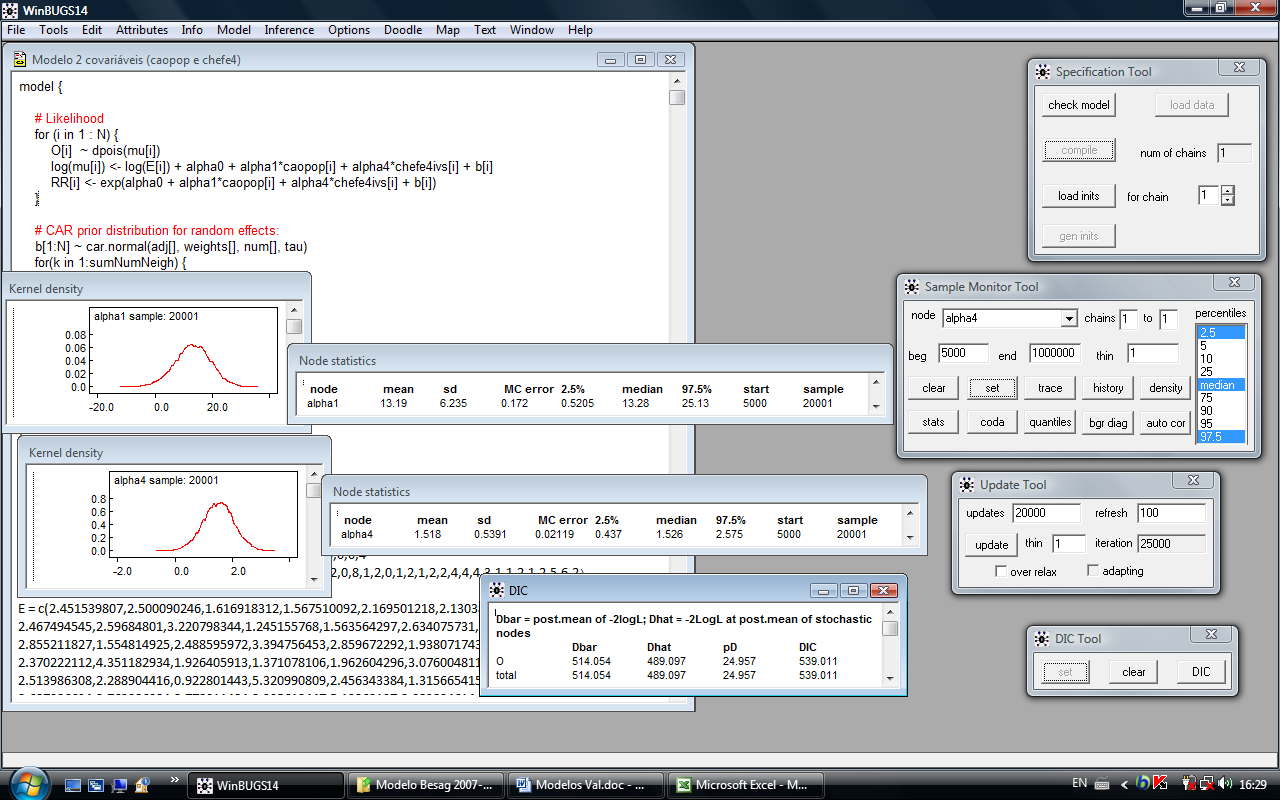


**Model 4**

alpha 1 e alpha 5 = number of infected dogs per inhabitant and percentage of householder with an income fewer than twice the Brazilian minimum wage (US$ 200.00)


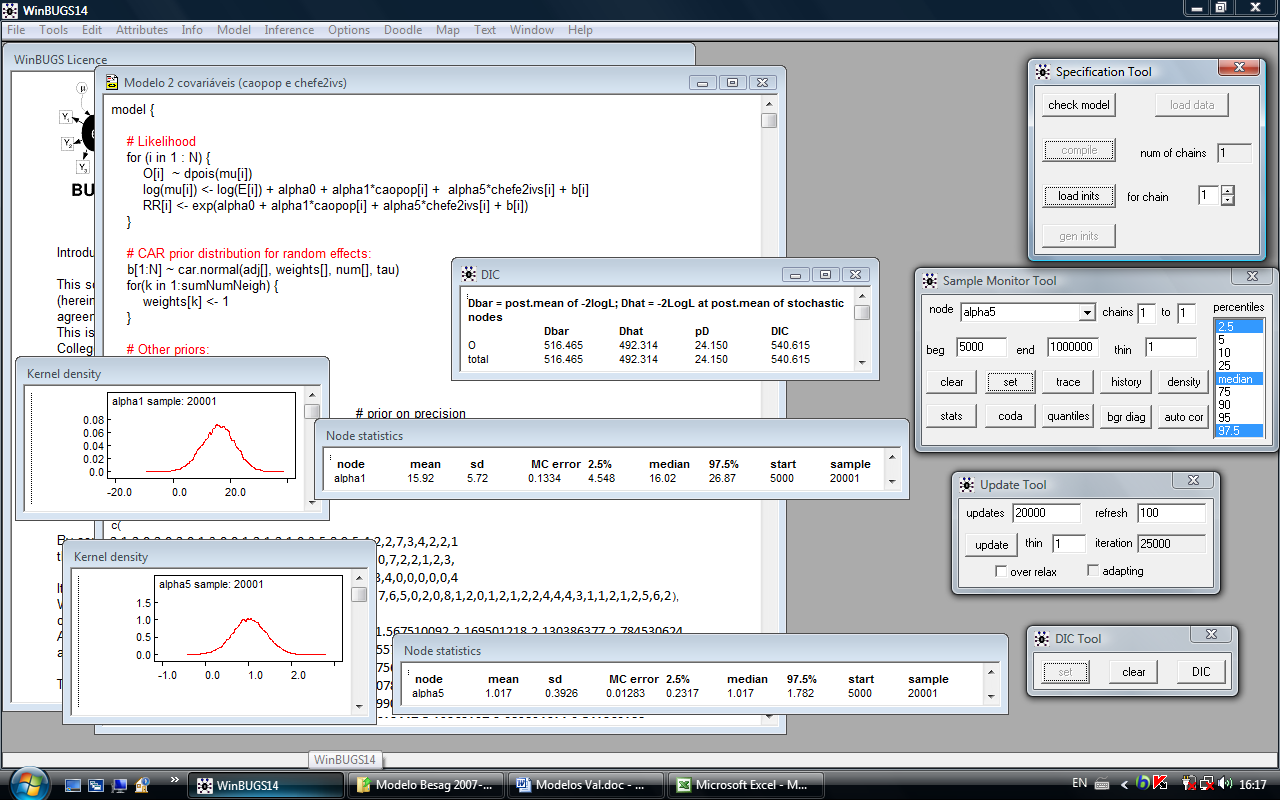

Supplement: Model S1 — Spatial statistical modeling: script and results of the models S1. (DOC) [file pntd.0002540.s001.doc]
